# Epidemiological and clinical characteristics of death from hemorrhagic fever with renal syndrome: a meta-analysis

**DOI:** 10.3389/fmicb.2024.1329683

**Published:** 2024-04-04

**Authors:** Wei Lu, Lin Kuang, Yuxing Hu, Jialing Shi, Qi Li, Wen Tian

**Affiliations:** ^1^School of Integrated Chinese and Western Medicine, Hunan University of Chinese Medicine, Changsha, China; ^2^College of Chinese Medicine, Beijing University of Chinese Medicine, Beijing, China

**Keywords:** hemorrhagic fever with renal syndrome, hantavirus, death patients, epidemiological features, clinical features, meta-analysis

## Abstract

**Introduction:**

Hemorrhagic fever with renal syndrome (HFRS) is an acute infectious disease comprising five stages: fever, hypotension, oliguria, diuresis (polyuria), and convalescence. Increased vascular permeability, coagulopathy, and renal injury are typical clinical features of HFRS, which has a case fatality rate of 1–15%. Despite this, a comprehensive meta-analyses of the clinical characteristics of patients who died from HFRS is lacking.

**Methods:**

Eleven Chinese- and English-language research databases were searched, including the China National Knowledge Infrastructure Database, Wanfang Database, SinoMed, VIP Database, PubMed, Embase, Scopus, Cochrane Library, Web of Science, Proquest, and Ovid, up to October 5, 2023. The search focused on clinical features of patients who died from HFRS. The extracted data were analyzed using STATA 14.0.

**Results:**

A total of 37 articles on 140,295 patients with laboratory–confirmed HFRS were included. Categorizing patients into those who died and those who survived, it was found that patients who died were older and more likely to smoke, have hypertension, and have diabetes. Significant differences were also observed in the clinical manifestations of multiple organ dysfunction syndrome, shock, occurrence of overlapping disease courses, cerebral edema, cerebral hemorrhage, toxic encephalopathy, convulsions, arrhythmias, heart failure, dyspnea, acute respiratory distress syndrome, pulmonary infection, liver damage, gastrointestinal bleeding, acute kidney injury, and urine protein levels. Compared to patients who survived, those who died were more likely to demonstrate elevated leukocyte count; decreased platelet count; increased lactate dehydrogenase, alanine aminotransferase, and aspartate aminotransferase levels; prolonged activated partial thromboplastin time and prothrombin time; and low albumin and chloride levels and were more likely to use continuous renal therapy. Interestingly, patients who died received less dialysis and had shorter average length of hospital stay than those who survived.

**Conclusion:**

Older patients and those with histories of smoking, hypertension, diabetes, central nervous system damage, heart damage, liver damage, kidney damage, or multiorgan dysfunction were at a high risk of death. The results can be used to assess patients’ clinical presentations and assist with prognostication.

**Systematic review registration:**https://www.crd.york.ac.uk/prospero/, (CRD42023454553).

## 1 Introduction

Hemorrhagic fever with renal syndrome (HFRS) is an acute zoonotic infectious disease transmitted by rodents and is mainly caused by the *Orthohantavirus* genus of the Bunyavirus order *Bunyavirales* (Jonsson et al., 2010). More than 50 hantaviruses have been identified, among which Old World hantaviruses such as the Hantaan virus (HTNV), Dobrava-Belgrade virus (DOBV), Seoul virus (SEOV), Amur virus, and Puumala virus (PUUA) viruses are known to cause HFRS (Zuo et al., 2011; Jiang et al., 2017; Laenen et al., 2019).

Orthohantavirus has a single-stranded negative-sense RNA composed of an S fragment that encodes and forms a nucleocapsid protein, an M fragment that encodes a glycoprotein precursor, and an L fragment that is synthetically dependent on RNA polymerase and other reverse transcriptase (Afzal et al., 2023). It mainly attacks the vascular endothelial cells of the human body, resulting in multiorgan cell damage; however, the pathogenesis of the disease is not fully understood (Vaheri et al., 2013a; Manigold and Vial, 2014). Moreover, the clinical symptoms of HFRS caused by different viruses are different. PUUV causes mild HFRS symptoms, SEOV causes moderate symptoms, and HTNV and DOBV cause severe HFRS symptoms (Sehgal et al., 2023).

HFRS first attracted attention in North Korea in 1953. In 1978, the virus was isolated by scholars and officially named HTNV (Jiang et al., 2017). HFRS is endemic in Europe and Asia, with approximately 100,000 HFRS cases were reported each year, 90% of which occur in China (Vaheri et al., 2013a). Its clinical features include acute kidney injury, severe thrombocytopenia, coagulopathy, bleeding, and flu-like symptoms, which can manifest as mild, severe, or critical (Tariq and Kim, 2022). Clinically, the disease comprises five phases: fever, hypotension, oliguria, diuresis (polyuria), and convalescence (Jiang et al., 2016). During the febrile phase, the patient presents with nonspecific symptoms, including fever, chills, nausea and vomiting, and headache (Sehgal et al., 2023). Symptoms of hypotension, leukocytosis, and thrombocytopenia predominate during the hypotensive phase, and shock can also occur in patients with severe disease (Sehgal et al., 2023). The oliguric phase presents with reduced urine output or even anuria, hematuria, or proteinuria. Patients are at increased risk of acute renal injury, and > 50% of patients die in this period (Sehgal et al., 2023). In the polyuria phase, there is an increase in urine output and recovery from kidney damage, while in the recovery phase, symptoms such as fatigue and weakness are recovered (Jiang et al., 2016; Sehgal et al., 2023). HFRS has a high case fatality rate ranging 1–15%, with PUUA mainly caused in Europe and HTNV in Asia (Vaheri et al., 2013b; Shirai et al., 2016).

HFRS is a zoonotic disease transmitted through contact with water sources, food, and aerosols contaminated with rodent feces, urine, saliva, or direct bites (Douron et al., 1984; Shirai et al., 2016). Human-to-human transmission has also been reported (Vaheri et al., 2013a). Hantavirus is unusually stable and can survive at room temperature for over 10 days (Kallio et al., 2006; Hardestam et al., 2007). Unfortunately, the disease currently lacks specific antiviral therapies or vaccines and can only be treated with supportive care (Zou et al., 2016).

HFRS is associated with high mortality; however, there exists little summative evidence-based research on the epidemiology and clinical characteristics of HFRS-induced deaths. Therefore, this article presents a meta-analysis of the epidemiology and clinical symptoms of patients who died from HFRS. The findings may help improve clinical understanding and ability to treat patients with HFRS.

## 2 Methods

### 2.1 Data sources and searches

**Figure 1:**
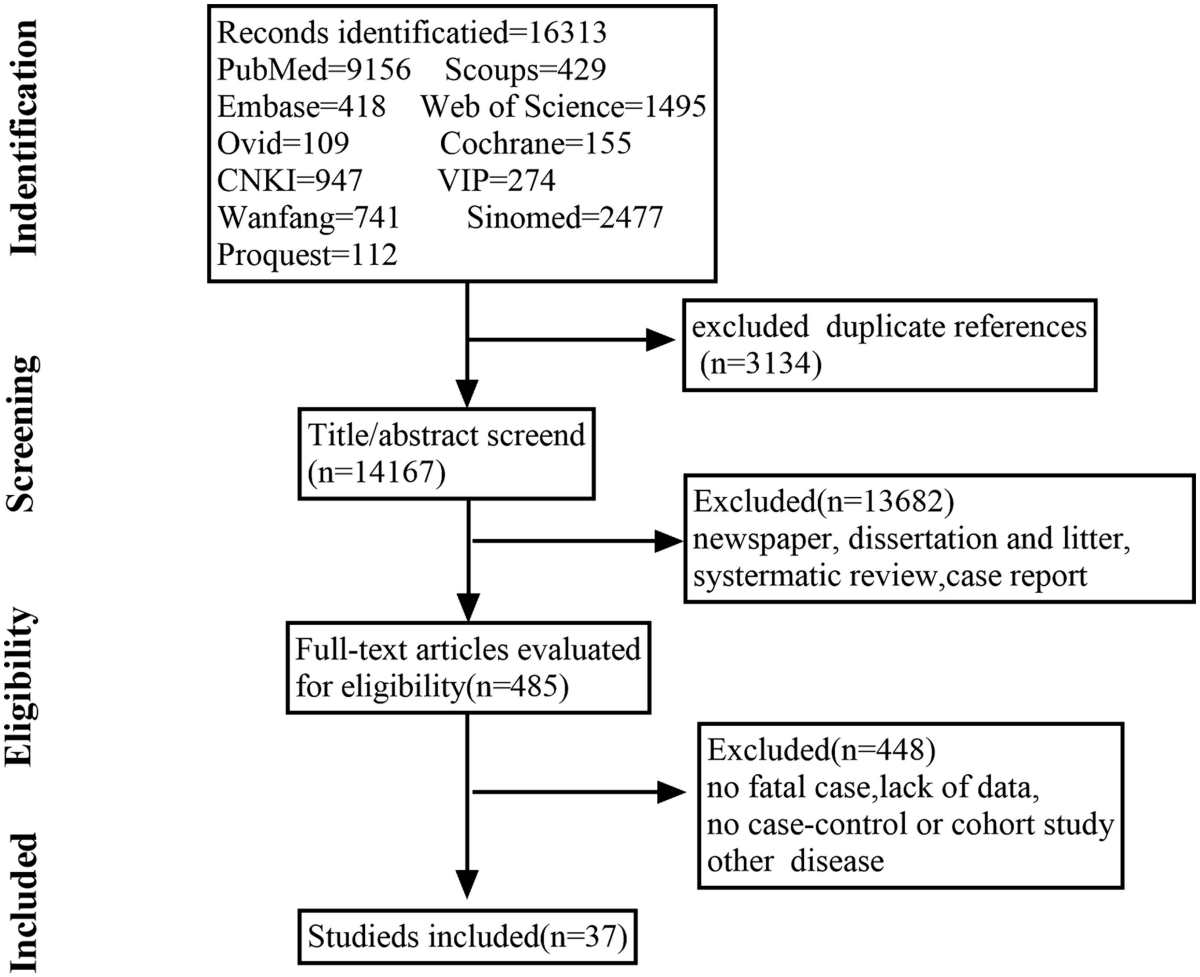
Flowchart of the literature screening process.

On October 5, 2023, English-and Chinese-language databases, including PubMed, Scopus, Embase, Web of Science, Proquest, Cochrane Library, Ovid, China National Knowledge Infrastructure Database, VIP Database, Wanfang Database, and SinoMed, were carefully searched. Using search strings such as “Hemorrhagic Fever with Renal Syndrome,” “HFRS,” “Hantavirus,” “Epidemic Hemorrhagic Fever,” and “Korean Hemorrhagic Fever.” The literature search process and results are shown in Figure 1. The study was registered to the PROSPERO platform (CRD42023454553).

### 2.2 Selection criteria

To be included in this meta-analysis, the study had to feature patients that met one or more of the following diagnostic criteria: (1) serum-specific IgM positive or serum-specific IgG titers were more than four times higher during convalescence than during the acute phase, or hantavirus RNA was detected from patient specimens or isolation of hantavirus from patient specimens; (2) the study must be prospective or retrospective; (3) study results should include the total number of deaths and survivors and must provide laboratory data for the deceased and survival groups, respectively. Additionally, (1) non-Chinese or non-English studies, (2) conferences and literature reviews, and (3) duplicate publications were excluded.

### 2.3 Data extraction and quality assessment

Two independent reviewers (Lu and Tian) carefully reviewed and selected all included articles separately. In cases where the inclusion or exclusion of a particular study was in question, the reviewers reviewed the publication and arrived at a consensus decision. If unable to agree, the final decision was made by the third author/reviewer (Kuang).

For all available studies, two independent reviewers evaluated the quality of the included studies using the Newcastle–Ottawa Scale (NOS). Then, using the sorted table, the two jointly extracted data about authors, years, sample size, the patients’ epidemiological and personal histories, signs and symptoms, laboratory test results, treatment, and final length of hospital stay from all included studies. Articles with available full text were carefully read before selection for inclusion. Disputes over the data in this study were resolved at the discretion of the third reviewer (Kuang).

### 2.4 Meta–analysis

Statistical analysis was performed using Stata 14.0 software. Continuous data are presented as standardized mean differences (SMDs), categorical data are expressed as odds ratios (ORs), and 95% confidence intervals (CIs). Cochran’s Q and *I*^2^ tests were used for heterogeneity assessment as follows:

$$Q=\overset{K}{\underset{i=1}{\sum }}w_{i}{\left(x_{i}-{\bar{x}}_{w}\right)}^{2}$$

Here, x_i_ represents study I, s_i_ (i = 1, 2…, k).s_i_ represents the corresponding estimation standard error, x̄_w_ represents the reciprocal of the x_i_ variance estimate, and w_i_ = 1∕s_i_^2^. *I*^2^ was calculated as follows: *I*^2^ = 100% × (Q − df)/Q, where Q denotes Cochran’s Q heterogeneity statistic, and df denotes the degree of freedom (Hoaglin, 2016). Because a value of 0 is considered when *I*^2^ is negative, the value of *I*^2^ ranges between 0 and 100%. A *p*–value of >0.1 and *I*^2^ of <50% indicate insignificant between-study heterogeneity, a fixed-effect model was subsequently used for analysis. Otherwise, sensitivity and subgroup analyses were used to determine the source of the heterogeneity. If the heterogeneity could not be resolved, a random-effects model was used. Funnel plots were used to indicate publication bias.

## 3 Results

### 3.1 Literature search, basic information, and quality assessment

A total of 16,313 candidate studies were identified. After removing those that did not meet the inclusion criteria, the full text of 485 articles was read. Finally, 37 articles were included in this study, resulting in a total sample size of 140, 295 cases. The quality of all included studies was scored using the NOS scale. Information on the studies and NOS scale scores are detailed in Table 1. All included studies were ≥ 6 stars.

**Table 1:** Basic information and quality assessment of the included literature.

Author	Year	Type	Survival	Death	NOS			
					Selection	Comparability	Outcome	Scores
Zhu et al. (2014)	2014	Case–control study	171	31	☆☆☆	☆☆☆	☆☆	8☆
Wang et al. (2023)	2023	Case–control study	233	75	☆☆☆	☆☆☆	☆☆☆	9☆
Wu et al. (1996)	1996	Case–control study	13	8	☆☆	☆☆	☆☆	6☆
Li et al. (2017)	2017	Case–control study	90	4	☆☆	☆☆	☆☆	6☆
Tang et al. (2005)	2005	Case–control study	93	10	☆☆☆	☆☆	☆☆	7☆
Tian and Li (2009)	2009	Case–control study	72	16	☆☆☆	☆☆	☆☆☆	8☆
Fan (2000)	2000	Case–control study	129	41	☆☆	☆☆	☆☆	6☆
Zong and Li (2001)	2001	Case–control study	160	9	☆☆	☆☆	☆☆	6☆
Du et al. (2023)	2023	Case–control study	78	13	☆☆☆	☆☆☆	☆☆☆	9☆
Tian et al. (2022)	2022	Case–control study	137	12	☆☆☆	☆☆☆	☆☆☆	9☆
Du et al. (2021)	2021	Case–control study	91	14	☆☆☆	☆☆☆	☆☆☆	9☆
Du et al., (2014d)	2014	Cohort study	46	29	☆☆☆	☆☆☆	☆☆☆	9☆
Fan et al. (2018)	2018	Case–control study	133	13	☆☆☆	☆☆☆	☆☆☆	9☆
Xiong and He (2006)	2006	Case–control study	180	33	☆☆☆	☆☆	☆☆☆	8☆
Che et al. (2022)	2022	Case–control study	359	14	☆☆☆	☆☆☆	☆☆☆	9☆
Li et al. (2016)	2016	Case–control study	107	22	☆☆☆	☆☆☆	☆☆☆	9☆
Pan et al. (2001)	2001	Case–control study	127	32	☆☆☆	☆☆	☆☆	7☆
Du et al. (2014b)	2014	Case–control study	27	21	☆☆☆	☆☆☆	☆☆☆	9☆
Ma et al. (2018)	2018	Case–control study	116	19	☆☆☆	☆☆☆	☆☆☆	9☆
Willemann and Oliveira (2014)	2014	Case–control study	281	158	☆☆☆	☆☆☆	☆☆☆	9☆
Luo et al. (2006)	2006	Case–control study	93	14	☆☆☆	☆☆☆	☆☆☆	9☆
Yang (2022)	2022	Case–control study	458	12	☆☆☆	☆☆	☆☆☆	8☆
Zhang et al. (2015)	2015	Case–control study	375	85	☆☆☆	☆☆	☆☆	7☆
Hu et al. (2023)	2023	Case–control study	2,113	132	☆☆☆	☆☆	☆☆☆	8☆
Li (2016)	2016	Case–control study	274	51	☆☆☆	☆☆☆	☆☆☆	9☆
Guo et al. (2012)	2012	Case–control study	90	30	☆☆☆	☆☆	☆☆☆	8☆
Du et al. (2014b)	2014	Case–control study	46	31	☆☆☆	☆☆	☆☆☆	8☆
Ye (2003)	2003	Case–control study	26	5	☆☆	☆☆	☆☆☆	7☆
Shang et al. (2009)	2009	Case–control study	78	19	☆☆	☆☆☆	☆☆☆	8☆
Yang (2012)	2012	Case–control study	45	7	☆☆☆	☆☆	☆☆	7☆
Qin (2015)	2015	Case–control study	26	10	☆☆	☆☆	☆☆	6☆
Xiong et al. (2012)	2012	Case–control study	348	21	☆☆	☆☆	☆☆	6☆
Fu and Yang, (2014)	2014	Case–control study	122,085	1,391	☆☆☆	☆☆	☆☆☆	8☆
Liu et al. (2017)	2017	Case–control study	1,536	7	☆☆☆	☆	☆☆☆	7☆
Du et al. (2014c)	2014	Case–control study	29	26	☆☆	☆☆	☆☆	6☆
Wu et al. (2014)	2014	Case–control study	7,220	78	☆☆☆	☆☆	☆☆	7☆
He et al. (2023)	2023	Case–control study	302	15	☆☆☆	☆☆☆	☆☆	8☆

### 3.2 Sex

A total of 16 articles contain sex-based analyses. The heterogeneity test revealed an *I*^2^ of 0.00% and *p*-value of <0.1, indicating low heterogeneity between patients who died and those who survived. A fixed-effect model was chosen for this study. Ultimately, no significant sex-based differences were found between the two groups (OR = 0.991, 95% CI: 0.809–1.212, *p* = 0.927; Supplementary Figure S1A). The funnel plot appeared symmetric after the decompression of publication bias (Supplementary Figure S1B).

### 3.3 Age

On examining the 11 studies that collected age data, *I*^2^ was 74% and the *p*-value was <0.1. A random-effects model revealed that patients who died tended to be older than those who survived (SMD = 0.478, 95% CI: 0.21–0.745, *p* < 0.001; Supplementary Figure S2A). After grouping the total number of cases in the included studies, subgroup analysis showed that heterogeneity was mainly attributable to studies with a total number greater than 300 (Supplementary Figure S2B). In the nine studies that included patients >60 years, the heterogeneity test revealed an *I*^2^ of 47.8 and *p*-value of <0.1, indicating some heterogeneity in the selected literature (Supplementary Figure S2C). The sensitivity analyses indicated that the Fu et al. study may have been a major source of the observed heterogeneity (Fu and Yang, 2014; Supplementary Figure S2D). After removing this study, patients who died were found to be 2.409 times more likely to be aged >60 years compared to those who survived (95% CI: 1.895–3.063, *p* < 0.001; Supplementary Figure S2E).

### 3.4 Epidemiology and personal history

Five studies examined onset season (whether the onset occurred in October through December) and found no significant between-group difference (OR = 1.194, 95% CI: 0.699–2.039, *p* = 0.517; Supplementary Figure S3A). In the eight articles that included data on patient occupation (farmer or non-farmer), there were no significant between-group differences (OR = 1.047, 95% CI: 0.522–2.098, *p* = 0.897; Supplementary Figure S3B). In the study on patients’ personal histories, significantly more patients who died were smokers than those who survived [the smoking rate was 1.357 times higher among those who died than among those who survived (95% CI: 1.030–1.787, *p* = 0.030; Supplementary Figure S3C)]. There was no significant between-group difference in alcohol consumption (OR = 1.007, 95% CI: 0.715–1.42, *p* = 0.966; Supplementary Figure S3D).

### 3.5 Hospital admissions

Basic information collected from patients on admission included their temperature at admission and the onset time. A total of seven studies analyzed the time from onset to hospital stay. Using a fixed-effect model, it was found that the onset time among patients who died was lower than that among those who survived (SMD = −0.391, 95% CI: −0.521 to −0.260, *p* < 0.001; Supplementary Figure S4A). However, four studies on body temperature at admission found no significant difference between the two groups (SMD = −0.006, 95% CI: −0.202 to 0.190, *p* = 0.951; Supplementary Figure S4B).

### 3.6 Comorbidities

The patients’ main comorbidities included diabetes, hypertension, and coronary heart disease. Five articles included data on patients with diabetes, and the incidence of comorbid diabetes was 1.991 times higher among patients who died than among those who survived (95% CI: 1.080–3.670, *p* = 0.027; Supplementary Figure S5A). Five studies collected data on hypertension; the incidence of hypertension was 2.811 times higher among patients who died than among those who survived (95% CI: 2.009–3.933, *p* < 0.001; Supplementary Figure S5B). Three studies who examined patients with coronary disease were included, and no significant between-group differences were observed (OR = 6.849, 95% CI: 0.547–76.951, *p* = 0.138; Supplementary Figure S5C).

### 3.7 Clinical manifestations

Four studies collected data on multiple organ dysfunction syndrome (MODS). Patients who died were 76.93 times more likely to demonstrate MODS complications (95% CI: 12.689–466.432, *p* < 0.001; Supplementary Figure S6A) than those who survived. A total of four studies analyzed shock symptoms, and it was found that shock was 24.075 times more likely in patients who died than in those who survived (95% CI: 8.941–64.825, *p* < 0.001; Supplementary Figure S6B). Four studies described overlapping disease courses, and it was found that the probability of death was 4.412 times higher than that of survival (95% CI: 1.744–11.158, *p* = 0.002; Supplementary Figure S6C). Eleven studies collected data on bacteremia; there was no significant between-group difference in the incidence of concurrent bacteremia (OR = 1.997, 95% CI: 0.953–4.185, *p* = 0.067; Supplementary Figure S6D).

In studies involving patients with craniocerebral injuries, six analyzed symptoms of cerebral edema. Patients who died had 12.566 times higher incidence of cerebral edema than those who survived (95% CI: 4.428–35.659, *p* < 0.001; Supplementary Figure S7A). Three studies analyzed toxic encephalopathy, and patients who died had 9.71 times higher incidence than those who survived (95% CI: 2.847–33.12, *p* < 0.001; Supplementary Figure S7B). Three studies analyzed symptoms of brain hemorrhage; the incidence was 88.652 times higher among patients who died than among those who survived (95% CI: 31.454–249.863, *p* < 0.001; Supplementary Figure S7C). In three studies that analyzed twitch, the incidence was 9.341 times higher among those who died than among those who survived (95% CI: 1.721–50.397, *p* = 0.010; Supplementary Figure S7D).

Among patients with concurrent cardiac injuries, four had arrhythmia. Patients who died had a 4.337 times higher incidence of arrhythmia than those who survived (95% CI: 1.046–18.307, *p* = 0.043; Supplementary Figure S8A). Further, the incidence of heart failure was 5.55 times higher among those who died than among those who survived (95% CI = 3.638–9.609, *p* < 0.001; Supplementary Figure S8B).

Four studies analyzed patients with symptoms of lung injury. The incidence of dyspnea was 2.916 times higher (95% CI: 1.377–6.176, *p* = 0.005; Supplementary Figure S9A), the incidence of acute respiratory distress syndrome (ARDS) was 19.068 times higher (95% CI: 8.105–44.86, *p* < 0.001; Supplementary Figure S9B), and the incidence of lung infections was 3.58 times higher among those who died than among those who survived (95% CI: 1.399–9.158, *p* = 0.008; Supplementary Figure S9C).

Four studies analyzed digestive complications—including liver injury; here, the mortality rate was 3.905 times higher among those who died than among those who survived (95%CI: 1.98–7.703, *p* < 0.001; Supplementary Figure S10A). Of the 10 studies that analyzed gastrointestinal bleeding, the incidence was 2.784 times higher among those who died than among those who survived (95% CI: 1.602–4.839, *p* < 0.001; Supplementary Figure S10B).

Five studies on urinary system injuries included patients with acute kidney injury (AKI). The incidence of acute kidney injury was 0.323 times lower among those who died (95% CI: 0.114–0.911, *p* = 0.033; Supplementary Figure S11A) than among those who survived. Three studies analyzed urine protein levels; patients who died were 2.117 times more likely to demonstrate abnormal urine protein levels (95% CI: 1.602–4.839, *p* < 0.001; Supplementary Figure S11B) than those who survived.

### 3.8 Laboratory test results

Eleven studies analyzed white blood cell counts (WBC); elevated WBC counts were more common among patients who died (SMD = 0.717, 95% CI: 0.279–1.154, *p* = 0.001; Supplementary Figure S12A) than among those who survived. Thirteen studies analyzed platelet counts; here, patients who died were more likely to demonstrate reduced platelet counts (SMD = −1.072, 95% CI: −1.806 to −0.377, *p* = 0.004; Supplementary Figure S12B) than those who survived. Six studies analyzed hemoglobin levels, collectively revealing no significant between-group differences (SMD = −0.157, 95% CI: −0.586 to 0.273, *p* = 0.475; Supplementary Figure S12C).

Three studies assessed lactate dehydrogenase levels. Patients who died were more likely to have elevated lactate dehydrogenase levels (SMD = 2.015, 95% CI: 0.524–3.506, *p* = 0.008; Supplementary Figure S13A) than those who survived. Similar results were found for aspartate aminotransferase (AST) (SMD = 1.067, 95% CI: 0.645–1.490, *p* < 0.001; Supplementary Figure S13B) and alanine aminotransferase (ALT) levels (SMD = 0.829, 95% CI: 0.258–0.936, *p* = 0.002; Supplementary Figure S13C). Five studies analyzed prothrombin time (PT), revealing that PT prolongation was more common among patients who died (SMD = 1.145, 95% CI: 0.426–1.827, *p* < 0.001; Supplementary Figure S13D) than among those who survived. Five studies analyzed activated partial thromboplastin time (APTT). Here, patients who died had significantly higher APTT levels than those who survived (SMD = 1.154, 95% CI: 0.436–1.873, *p* = 0.002; Supplementary Figure S13E). Nine studies included data on albumin levels. Patients who died had lower albumin levels than those who survived (SMD = −0.574, 95% CI: −0.855 to −0.293, *p* < 0.001; Supplementary Figure S13F). Patients who died were significantly more likely to demonstrate lower chloride ions (SMD = −0.449, 95% CI: −0.875 to −0.024, *p* = 0.038; Supplementary Figure S13G) and fibrinogen levels (SMD = −0.626, 95% CI: −0.8285 to −0.242, *p* < 0.001; Supplementary Figure S13H) than those who survived.

Across all examined studies, no significant differences were found in urea nitrogen (SMD = 0.362, 95% CI: −0.016 to 0.741, *p* = 0.061; Supplementary Figure S14A), serum creatinine (SMD = −0.007, 95% CI: −1.146 to 1.133, *p* = 0.991; Supplementary Figure S14B), sodium ions (SMD = −0.537, 95% CI: −1.09 to 0.015, *p* = 0.057; Supplementary Figure S14C), potassium ions (SMD = 1.147, 95% CI: −0.475 to 2.769, *p* = 0.166; Supplementary Figure S14D), and total bilirubin (SMD = 0.454, 95% CI: −0.028 to 0.936, *p* = 0.065; Supplementary Figure S14E) levels between those who died with those who survived.

### 3.9 Treatment

Interestingly, patients who died used more continuous renal replacement therapy (OR = 4.487, 95% CI: 1.186–16.981, *p* = 0.027; Supplementary Figure S15A) and less dialysis than those who survived (OR = 0.304, 95% CI: 0.201–0.459, *p* < 0.001; Supplementary Figure S15B). Four studies noted rates of mechanical ventilation, identifying no significant between-group differences (OR = 4.709, 95% CI: 0.471–47.118, *p* = 0.187; Supplementary Figure S15C).

### 3.10 Hospitalization

Nine studies included length of hospital stay as a dependent variable, with *I*^2^ > 50% and *p*-value <0.1. Consequently, a random-utility model was used to determine that patients who died had a shorter average length of hospital stay than those who survived (SMD = −1.426, 95% CI: −2.168 to −0.685, *p* < 0.001; Supplementary Figure S16A). The source of this heterogeneity comprised literature published between 2018 and 2022 after subgroup analysis (Supplementary Figure S16B).

## 4 Discussion

HFRS is an infectious disease mainly endemic to Asia and Europe, with China and South Korea being the main endemic regions (Jiang et al., 2016; Dong et al., 2019); however, its incidence throughout Europe and Asia is increasing. Consequently, HFRS is a serious public health concern in its endemic regions.

Although HFRS is more common in men than in women, women are at a higher risk of death from the condition (Klein et al., 2011). Surprisingly, we found no significant sex-based was found between patients who died and those who survived; however, older patients were at a higher risk of death. Fu et al.’s well-powered study found that patients aged >60 years were at a higher risk of death (Fu and Yang, 2014). The present study’s sensitivity analysis revealed considerable heterogeneity in results, possibly attributed to the large sample size of the Fu et al. study; however, the risk of death in patients aged >60 years remained high even after the removal of this study. This disparity may be related to older patients’ immune statuses, underlying medical conditions, and the prevalence of immunization among adolescents (He et al., 2013).

Studies that reported personal history data found that smokers were at a higher risk of death than nonsmokers. Other studies found smoking to be a risk factor for HFRS infection, given the ability of nicotine to increase oxidative stress and damage the kidneys (Laine et al., 2012; Arany et al., 2013; Tervo et al., 2015; Latronico et al., 2018). However, no significant between-group difference in alcohol consumption was observed, similar to the findings reported by Tervo et al. (2022). Mantula et al. (2017) showed that hyperglycemia is a risk factor for poor prognosis in patients with HFRS. In these studies, patients with diabetes were at a higher risk of death (Mantula et al., 2017; Tietavainen et al., 2019, 2021). It is hypothesized that this may be related to the ability of diabetes to increase endothelial dysfunction through oxidative stress (Karan et al., 2020). More patients with hypertension died, and studies have also shown that hypertension affects long-term prognosis in patients with HFRS (Latus et al., 2015), likely because of the association between vascular endothelial damage and aggravated hypertension.

Similar to Jiang et al.’s findings, patients who died were more likely to demonstrate multistage overlap (Jiang et al., 2016), including shock and multiorgan damage, central nervous system damage, and injuries to the lungs, heart, liver, and kidneys. These effects could be caused by the invading hantavirus’s effects on the endothelial cells of the host’s organs, eliciting a strong immune response (Liu et al., 2019). Patients who died also had a higher rate of ARDS than those who survived, potentially attributable to acute progressive noncardiogenic pulmonary edema (Zou et al., 2016).

Similarly, our studies on patients’ cardiovascular systems showed higher rates of heart failure and arrhythmias among those who died than among those who survived. A Swedish study also showed that cardiovascular disease frequently causes HFRS-related deaths (Connolly-Andersen et al., 2013). Patients in the concomitant death group were more likely to demonstrate proteinuria, typical of HFRS. Additionally, proteinuria is a known risk factor for HFRS complicated by acute pancreatitis (Wang et al., 2023).

Furthermore, laboratory analyses results revealed that patients who died had lower platelet counts than those who survived. This could have resulted from impaired platelet production, impaired thrombin aggregation during hantavirus infection, and hantavirus-guided resting platelets adhering to infected endothelial cells, leading to circulating thrombocytopenia (Gavrilovskaya et al., 2010; Laine et al., 2015). Thrombocytopenia is associated with HFRS and severe kidney damage and is frequently considered when diagnosing HFRS (Denecke et al., 2010). Similarly, patients who died had prolonged PT and APTT, potentially resulting from tissue factor upregulation caused by the inflammatory storm, leading to coagulation dysfunction and ultimately increasing endothelial cell permeability (Puhlmann et al., 2005). Among patients who died, elevated WBC count was associated with inflammation; the cytokine storm in HFRS patients could also lead to leukocyte extravasation by affecting the permeability of endothelial cells (Nourshargh and Alon, 2014). Decreased fibrinogen in patients with HFRS who died may be due to the lysis of prothrombin into fibrin monomers during coagulation (Koskela et al., 2021; Sehgal et al., 2023). Patients who died had higher AST and ALT levels than those who survived (routinely measured in patients with HFRS). Hantaviruses can invade multiple organ systems, and AST abnormalities are associated with a poorer prognosis (He et al., 2023).

In the present study, patients who died while receiving supportive care for HFRS were more likely to receive renal replacement therapy. Sargianou et al. showed that timely and correct supportive care improved survival with HFRS (Sargianou et al., 2012). Furthermore, continuous renal therapy was previously deemed suitable for patients with severe HFRS, those with multiorgan damage, and those with encephalopathy and other complications, consistent with the present study results (Jiang et al., 2016). Interestingly, it was found that AKI was more pronounced in patients who survived than in those who died. Outinen et al.’s (2015) study also reported that severe AKI is not a risk factor for HFRS-induced death in patients. This is because patients who died are more likely to have overlapping stages of fever, hypotension, and oliguria, and are in a critical condition that might result in death from other fatal comorbidities, such as ARDS (Du et al., 2014a). The overlapping phase of HFRS usually occurs early in the course of the disease, when acute renal failure, one of the severe stages of AKI, has not yet developed or been detected. Timely dialysis treatment can also improve the prognosis of patients with acute renal failure (Mehta et al., 2007; Zhu et al., 2014). In contrast, urine protein appears significantly earlier than acute kidney failure and has been shown to be a risk factor for the prognosis of HFRS (Mantula et al., 2017).

This study has several limitations that should be considered. First, only English and Chinese articles were reviewed; studies in Spanish, French, or other languages were not included, potentially introducing bias. Second, between-group comparisons of viral loads were lacking. Third, the study data were insufficient for meta-analyzing patients’ inflammatory factor storms, resulting in some heterogeneity in the study results, and the possibility of bias. Therefore, additional well-powered investigations are needed in the future.

## 5 Conclusion

The present meta-analysis results demonstrated a significant difference between patients who died and those who survived. Specifically, patients who died demonstrated more severe disease than those who survived. Patients with personal histories of smoking, diabetes, and high blood pressure were at a higher risk of death than those who survived. Patients who died also demonstrated more severe damage to the central nervous system, lungs, and heart, and significantly fewer platelets and higher urine protein levels than those who survived (Supplementary Table S1). These findings can be used to assess clinical practice methods and to ascertain prognosis.

## Data availability statement

The original contributions presented in the study are included in the article/Supplementary material, further inquiries can be directed to the corresponding author.

## Author contributions

WL: Writing – original draft. LK: Writing – review & editing. YH: Writing – review & editing, Methodology. JS: Methodology, Writing – review & editing. QL: Methodology, Writing – original draft. WT: Methodology, Writing – review & editing.

## References

[ref9001] Afzal S. Ali L. Batool A. Afzal M. Kanwal N. Hassan M. . (2023). Hantavirus: an overview and advancements in therapeutic approaches for infection. Front. Microbiol. 14:1233433. doi: 10.3389/fmicb.2023.1233433 37901807 PMC10601933

[ref1] Arany I. Clark J. Reed D. K. Juncos L. A. (2013). Chronic nicotine exposure augments renal oxidative stress and injury through transcriptional activation of p66shc. Nephrol. Dial. Transplant. 28, 1417–1425. doi: 10.1093/ndt/gfs596, PMID: 23328708 PMC3685305

[ref2] Che L. Wang Z. Du N. Li L. Zhao Y. Zhang K. . (2022). Evaluation of serum ferritin, procalcitonin, and c-reactive protein for the prediction of severity and mortality in hemorrhagic fever with renal syndrome. Front. Microbiol. 13:865233. doi: 10.3389/fmicb.2022.865233, PMID: 35677912 PMC9169039

[ref3] Connolly-Andersen A. M. Ahlm K. Ahlm C. Klingstrom J. (2013). Puumala virus infections associated with cardiovascular causes of death. Emerg. Infect. Dis. 19, 126–128. doi: 10.3201/eid1901.111587, PMID: 23260342 PMC3557968

[ref4] Denecke B. Bigalke B. Haap M. Overkamp D. Lehnert H. Haas C. S. (2010). Hantavirus infection: a neglected diagnosis in thrombocytopenia and fever? Mayo Clin. Proc. 85, 1016–1020. doi: 10.4065/mcp.2009.0040, PMID: 21037045 PMC2966365

[ref5] Dong Y. Ma T. Zhang X. Ying Q. Han M. Zhang M. . (2019). Incorporation of cd40 ligand or granulocyte-macrophage colony stimulating factor into hantaan virus (htnv) virus-like particles significantly enhances the long-term immunity potency against htnv infection. J. Med. Microbiol. 68, 480–492. doi: 10.1099/jmm.0.000897, PMID: 30657443

[ref6] Douron E. Moriniere B. Matheron S. Girard P. M. Gonzalez J. P. Hirsch F. . (1984). Hfrs after a wild rodent bite in the haute-savoie--and risk of exposure to hantaan-like virus in a Paris laboratory. Lancet 1, 676–677. doi: 10.1016/s0140-6736(84)92187-1 6142362

[ref7] Du H. Hu H. Li J. Wang X. Jiang H. Lian J. . (2023). High levels of exfoliated fragments following glycocalyx destruction in hemorrhagic fever with the renal syndrome are associated with mortality risk. Front Med (Lausanne) 10:1096353. doi: 10.3389/fmed.2023.1096353, PMID: 37138736 PMC10149802

[ref8] Du H. Hu H. Wang P. Wang X. Zhang Y. Jiang H. . (2021). Predictive value of pentraxin-3 on disease severity and mortality risk in patients with hemorrhagic fever with renal syndrome. BMC Infect. Dis. 21:445. doi: 10.1186/s12879-021-06145-0, PMID: 34001041 PMC8130374

[ref9] Du H. Li J. Jiang W. Yu H. Zhang Y. Wang J. . (2014a). Clinical study of critical patients with hemorrhagic fever with renal syndrome complicated by acute respiratory distress syndrome. PLoS One 9:e89740. doi: 10.1371/journal.pone.0089740, PMID: 24587001 PMC3933661

[ref10] Du H. Li J. Wang P. Bai X. (2014b). Application of renal replacement therapy in critical patients with hemorrhagic fever and renal syndrome. Med. J. Chin. Peoples Liber. Army 26, 42–45. doi: 10.3969/j.issn.2095-140X.2014.04.013

[ref11] Du H. Li J. Yu H. T. Rong Z. (2014c). Application of continuous renal replacement therapy and intermittent hemodialysis in patients with severe hemorrhagic fever with renal syndrome. Infect. Dis. Inf. 27, 18–21.

[ref12] Du H. Wang P. Z. Li J. Bai L. Li H. Yu H. T. . (2014d). Clinical characteristics and outcomes in critical patients with hemorrhagic fever with renal syndrome. BMC Infect. Dis. 14:191. doi: 10.1186/1471-2334-14-191, PMID: 24712579 PMC4020609

[ref13] Fan H. (2000). Analysis of factors related to secondary shock secondary to hemorrhagic fever with renal syndrome. Chin. Crit. Care Med. 12:239. doi: 10.3760/j.issn:1003-0603.2000.04.021

[ref14] Fan X. Deng H. Sang J. Li N. Zhang X. Han Q. . (2018). High serum procalcitonin concentrations in patients with hemorrhagic fever with renal syndrome caused by hantaan virus. Front. Cell. Infect. Microbiol. 8:129. doi: 10.3389/fcimb.2018.00129, PMID: 29868489 PMC5952221

[ref15] Fu M. Yang H. (2014). Analysis of epidemic haemorrhagic fever epidemic characteristics in China from 2004 to 2012. China Health Ind. 11, 35–36. doi: 10.16659/j.cnki.1672-5654.2014.28.042

[ref16] Gavrilovskaya I. N. Gorbunova E. E. Mackow E. R. (2010). Pathogenic hantaviruses direct the adherence of quiescent platelets to infected endothelial cells. J. Virol. 84, 4832–4839. doi: 10.1128/JVI.02405-09, PMID: 20181715 PMC2863738

[ref17] Guo N. Cui S. Wang X. Cui H. (2012). Role of hemorrhagic fever with renal syndrome(hfrs) critical score in predicting the prognosis for patients with hfrs. J. Shandong Univ. 50, 67–70. doi: 10.6040/j.issn.1671-7554.2012.11.014

[ref18] Hardestam J. Simon M. Hedlund K. O. Vaheri A. Klingstrom J. Lundkvist A. (2007). Ex vivo stability of the rodent-borne hantaan virus in comparison to that of arthropod-borne members of the bunyaviridae family. Appl. Environ. Microbiol. 73, 2547–2551. doi: 10.1128/AEM.02869-06, PMID: 17337567 PMC1855600

[ref19] He S. Han Q. Wang X. Zhang X. Li N. Liu Z. (2023). Aspartate aminotransferase to platelet ratio at admission can predict the prognosis of patients with hemorrhagic fever with renal syndrome. J. Med. Virol. 95:e29126. doi: 10.1002/jmv.29126, PMID: 37786231

[ref20] He X. Wang S. Huang X. Wang X. (2013). Changes in age distribution of hemorrhagic fever with renal syndrome: an implication of China’s expanded program of immunization. BMC Public Health 13:394. doi: 10.1186/1471-2458-13-394, PMID: 23622420 PMC3670207

[ref21] Hoaglin D. C. (2016). Misunderstandings about q and 'cochran's q test' in meta-analysis. Stat. Med. 35, 485–495. doi: 10.1002/sim.6632 26303773

[ref22] Hu H. F. Zhan J. Y. Du H. Yang Y. Y. (2023). Clinical epidemiological characteristics and prognostic risk factors in 2 245 patients with hemorrhagic fever with renal syndrome. Chin. J. Infect. Dis. 41, 70–76. doi: 10.3760/cma.j.cn311365-20220422-00141

[ref23] Jiang H. Du H. Wang L. M. Wang P. Z. Bai X. F. (2016). Hemorrhagic fever with renal syndrome: pathogenesis and clinical picture. Front. Cell. Infect. Microbiol. 6:1. doi: 10.3389/fcimb.2016.00001, PMID: 26870699 PMC4737898

[ref24] Jiang H. Zheng X. Wang L. Du H. Wang P. Bai X. (2017). Hantavirus infection: a global zoonotic challenge. Virol. Sin. 32, 32–43. doi: 10.1007/s12250-016-3899-x, PMID: 28120221 PMC6598904

[ref25] Jonsson C. B. Figueiredo L. T. Vapalahti O. (2010). A global perspective on hantavirus ecology, epidemiology, and disease. Clin. Microbiol. Rev. 23, 412–441. doi: 10.1128/CMR.00062-09 20375360 PMC2863364

[ref26] Kallio E. R. Klingstrom J. Gustafsson E. Manni T. Vaheri A. Henttonen H. . (2006). Prolonged survival of puumala hantavirus outside the host: evidence for indirect transmission via the environment. J. Gen. Virol. 87, 2127–2134. doi: 10.1099/vir.0.81643-0 16847107

[ref27] Karan A. Bhakkiyalakshmi E. Jayasuriya R. Sarada D. Ramkumar K. M. (2020). The pivotal role of nuclear factor erythroid 2-related factor 2 in diabetes-induced endothelial dysfunction. Pharmacol. Res. 153:104601. doi: 10.1016/j.phrs.2019.104601, PMID: 31838079

[ref28] Klein S. L. Marks M. A. Li W. Glass G. E. Fang L. Q. Ma J. Q. . (2011). Sex differences in the incidence and case fatality rates from hemorrhagic fever with renal syndrome in China, 2004-2008. Clin. Infect. Dis. 52, 1414–1421. doi: 10.1093/cid/cir232, PMID: 21628481 PMC3146012

[ref29] Koskela S. Makela S. Strandin T. Vaheri A. Outinen T. Joutsi-Korhonen L. . (2021). Coagulopathy in acute puumala hantavirus infection. Viruses 13:1553. doi: 10.3390/v13081553, PMID: 34452419 PMC8402851

[ref30] Laenen L. Vergote V. Calisher C. H. Klempa B. Klingstrom J. Kuhn J. H. . (2019). Hantaviridae: current classification and future perspectives. Viruses 11:788. doi: 10.3390/v11090788, PMID: 31461937 PMC6784073

[ref31] Laine O. Joutsi-Korhonen L. Lassila R. Koski T. Huhtala H. Vaheri A. . (2015). Hantavirus infection-induced thrombocytopenia triggers increased production but associates with impaired aggregation of platelets except for collagen. Thromb. Res. 136, 1126–1132. doi: 10.1016/j.thromres.2015.10.003, PMID: 26462407

[ref32] Laine O. Joutsi-Korhonen L. Makela S. Mikkelsson J. Pessi T. Tuomisto S. . (2012). Polymorphisms of pai-1 and platelet gp ia may associate with impairment of renal function and thrombocytopenia in puumala hantavirus infection. Thromb. Res. 129, 611–615. doi: 10.1016/j.thromres.2011.11.007, PMID: 22133274 PMC3879723

[ref33] Latronico F. Maki S. Rissanen H. Ollgren J. Lyytikainen O. Vapalahti O. . (2018). Population-based seroprevalence of puumala hantavirus in Finland: smoking as a risk factor. Epidemiol. Infect. 146, 367–371. doi: 10.1017/S0950268817002904, PMID: 29310747 PMC9134512

[ref34] Latus J. Schwab M. Tacconelli E. Pieper F. M. Wegener D. Dippon J. . (2015). Clinical course and long-term outcome of hantavirus-associated nephropathia epidemica, Germany. Emerg. Infect. Dis. 21, 76–83. doi: 10.3201/eid2101.140861, PMID: 25533268 PMC4285283

[ref35] Li Q. (2016). Analysis of prognostic factors influencing patients with hemorrhagic fever with renal syndrome and early warning intervention countermeasures. Chin. J. Control Endem. Dis. 31, 690–691.

[ref36] Li S. Tian Y. Zhao G. Ma J. (2016). Analysis on risk factors of 22 cases of hemorrhagic fever with renal syndrome. China Mod. Doct. 54, 81–84.

[ref37] Li X. Zhang S. Cao H. H. (2017). Relationship between blood changes and prognosis in patients with hemorrhagic fever with renal syndrome. J. Trop. Med. 17, 772–775. doi: 10.3969/j.issn.1672-3619.2017.06.021

[ref38] Liu J. Deng A. Peng Z. (2017). Analysis of epidemiologic characteristics of hemorrhagic fever patients with renal syndrome (hfrs) in Guangdong from 2009 to 2013. J. Med. Pest Control 33, 606–609. doi: 10.7629/yxdwfz201706005

[ref39] Liu R. Ma H. Shu J. Zhang Q. Han M. Liu Z. . (2019). Vaccines and therapeutics against hantaviruses. Front. Microbiol. 10:2989. doi: 10.3389/fmicb.2019.02989, PMID: 32082263 PMC7002362

[ref40] Luo R. Chen Y. Zhang Y. (2006). Prognosis of hemorrhagic fever with renal syndrome used by roc curves and discriminant analysis. Chin. J. Zoonoses 5, 481–482. doi: 10.3969/j.issn.1002-2694.2006.05.028

[ref41] Ma X. Li F. Yang H. Wang F. Zhao H. (2018). The timing of treatment and prognostic factors by continuous renai repiacement therapy in severe hemorrhagic fever with renai svndrome. China Trop. Med. 20, 927–930. doi: 10.1186/s12879-020-05638-8, PMID: 33272200 PMC7713152

[ref42] Manigold T. Vial P. (2014). Human hantavirus infections: epidemiology, clinical features, pathogenesis and immunology. Swiss Med. Wkly. 144:w13937. doi: 10.4414/smw.2014.13937, PMID: 24652684

[ref43] Mantula P. S. Outinen T. K. Clement J. Huhtala H. Porsti I. H. Vaheri A. . (2017). Glomerular proteinuria predicts the severity of acute kidney injury in puumala hantavirus-induced tubulointerstitial nephritis. Nephron 136, 193–201. doi: 10.1159/000459634, PMID: 28319945

[ref44] Mehta R. L. Kellum J. A. Shah S. V. Molitoris B. A. Ronco C. Warnock D. G. . (2007). Acute kidney injury network: report of an initiative to improve outcomes in acute kidney injury. Crit. Care 11:R31. doi: 10.1186/cc5713, PMID: 17331245 PMC2206446

[ref45] Nourshargh S. Alon R. (2014). Leukocyte migration into inflamed tissues. Immunity 41, 694–707. doi: 10.1016/j.immuni.2014.10.008, PMID: 25517612

[ref46] Outinen T. K. Makela S. Clement J. Paakkala A. Porsti I. Mustonen J. (2015). Community acquired severe acute kidney injury caused by hantavirus-induced hemorrhagic fever with renal syndrome has a favorable outcome. Nephron 130, 182–190. doi: 10.1159/000433563, PMID: 26139246

[ref47] Pan T. Xiu T. Gong Q. Zhang S. C. (2001). Analysis of causes of death from hemorrhagic fever with renal syndrome in 32 cases. Jiangsu Med. J. 27:465. doi: 10.3969/j.issn.0253-3685.2001.06.040

[ref48] Puhlmann M. Weinreich D. M. Farma J. M. Carroll N. M. Turner E. M. Alexander H. J. (2005). Interleukin-1beta induced vascular permeability is dependent on induction of endothelial tissue factor (tf) activity. J. Transl. Med. 3:37. doi: 10.1186/1479-5876-3-37, PMID: 16197553 PMC1276820

[ref49] Qin G. (2015). Clinical analysis of blood sodium and urea nitrogen values in oliguric phase in 169 patients with hemorrhagic fever. Yianbian Med. J. 43:44.

[ref50] Sargianou M. Watson D. C. Chra P. Papa A. Starakis I. Gogos C. . (2012). Hantavirus infections for the clinician: from case presentation to diagnosis and treatment. Crit. Rev. Microbiol. 38, 317–329. doi: 10.3109/1040841X.2012.673553, PMID: 22553984

[ref51] Sehgal A. Mehta S. Sahay K. Martynova E. Rizvanov A. Baranwal M. . (2023). Hemorrhagic fever with renal syndrome in asia: history, pathogenesis, diagnosis, treatment, and prevention. Viruses 15:561. doi: 10.3390/v15020561, PMID: 36851775 PMC9966805

[ref52] Shang Q. Yu J. Wang Y. (2009). Analysis of prognostic factors of severe renal syndrome. Pract. J. Med. Pharm. 26, 19–20. doi: 10.3969/j.issn.1671-4008.2009.01.009

[ref53] Shirai H. Yashima J. Tojimbara T. Honda K. (2016). Thrombotic microangiopathy caused by oral contraceptives in a kidney transplant recipient. Nephrology (Carlton) 21, 41–43. doi: 10.1111/nep.12769 26970708

[ref54] Tang Z. Ye X. Liao Y. (2005). Clinical observation of liver damages in epidemic hemorrhagic fever. J. First Mil. Med. Univ. 5, 593–594. doi: 10.3321/j.issn:1673-4254.2005.05.029 15897149

[ref55] Tariq M. Kim D. M. (2022). Hemorrhagic fever with renal syndrome: literature review, epidemiology, clinical picture and pathogenesis. Infec. Chemother. 54, 1–19. doi: 10.3947/ic.2021.0148, PMID: 35384417 PMC8987181

[ref56] Tervo L. Makela S. Syrjanen J. Huttunen R. Rimpela A. Huhtala H. . (2015). Smoking is associated with aggravated kidney injury in puumala hantavirus-induced haemorrhagic fever with renal syndrome. Nephrol. Dial. Transplant. 30, 1693–1698. doi: 10.1093/ndt/gfv273, PMID: 26150428 PMC4838005

[ref57] Tervo L. Outinen T. K. Makela S. Mustalahti J. Huhtala H. Porsti I. . (2022). Alcohol consumption and its influence on the clinical picture of puumala hantavirus infection. Viruses 14:500. doi: 10.3390/v14030500, PMID: 35336910 PMC8948946

[ref58] Tian X. Li H. (2009). Clinical analysis of the treatment of hemorrhagic fever with renal syndrome. J. Qiqihar Univ. Med. 30, 924–925. doi: 10.3969/j.issn.1002-1256.2009.08.015

[ref59] Tian Z. Yao N. Wu Y. Wang F. Zhao Y. (2022). Serum superoxide dismutase level is a potential biomarker of disease prognosis in patients with hemorrhagic fever with renal syndrome caused by the hantaan virus. BMC Infect. Dis. 22:446. doi: 10.1186/s12879-022-07394-3, PMID: 35538453 PMC9087930

[ref60] Tietavainen J. Makela S. Huhtala H. Porsti I. H. Strandin T. Vaheri A. . (2021). The clinical presentation of puumala hantavirus induced hemorrhagic fever with renal syndrome is related to plasma glucose concentration. Viruses 13:1177. doi: 10.3390/v13061177, PMID: 34202952 PMC8235586

[ref61] Tietavainen J. Mantula P. Outinen T. Huhtala H. Porsti I. H. Niemela O. . (2019). Glucosuria predicts the severity of puumala hantavirus infection. Kidney Int. Rep. 4, 1296–1303. doi: 10.1016/j.ekir.2019.05.770, PMID: 31517148 PMC6734096

[ref62] Vaheri A. Henttonen H. Voutilainen L. Mustonen J. Sironen T. Vapalahti O. (2013a). Hantavirus infections in europe and their impact on public health. Rev. Med. Virol. 23, 35–49. doi: 10.1002/rmv.1722, PMID: 22761056

[ref63] Vaheri A. Strandin T. Hepojoki J. Sironen T. Henttonen H. Makela S. . (2013b). Uncovering the mysteries of hantavirus infections. Nat. Rev. Microbiol. 11, 539–550. doi: 10.1038/nrmicro3066, PMID: 24020072

[ref64] Wang T. Du H. Zhao Y. (2023). Analysis of clinical characteristics and construction of death risk model of severe patients with hemorrhagic fever with renal syndrome. Infect. Dis. Inf. 36, 43–50. doi: 10.3969/j.issn.1007-8134.2023.01.06

[ref65] Willemann M. C. Oliveira S. V. (2014). Risk factors associated with hantavirosis fatality: a regional analysis from a case-control study in Brazil. Rev. Soc. Bras. Med. Trop. 47, 47–51. doi: 10.1590/0037-8682-0243-2013, PMID: 24603736

[ref66] Wu W. Guo J. Q. Guan P. (2014). Analysis of epidemiological features of hemorrhagic fever with renal syndrome and associated environmental risk factors in Liaoning province, China during 2005-2007. Chin. J. Vector Biol. Control 25, 39–42. doi: 10.11853/j.issn.1003.4692.2014.01.011

[ref67] Wu A. H. Tan D. M. Ren P. S. (1996). Study of serum ldh and hbd levels in patients with hemorrhagic fever with renal syndrome. J. Central South Univ. 1, 75–77.

[ref68] Xiong X. Cheng H. Shi G. (2012). Analysis on death risk of severe cases of hemorrhagic fever with renal syndrome in gaoan city, Jiangxi province in 2010. Pract. Prev. Med. 19, 1650–1652. doi: 10.3969/j.issn.1006-3110.2012.11.015

[ref69] Xiong Q. He S. (2006). Univariate analysis affecting the prognosis of hemorrhagic fever with renal syndrome. J. Chin. Phys. 8, 537–538. doi: 10.3760/cma.j.issn.1008-1372.2006.04.059

[ref70] Yang L. (2012). Cases of 52 severe hemorrhagic fever with renal syndrome and multiple organ dysfunction syndrome analysis. Chin. J. Zoonoses 28, 410–411. doi: 10.3969/j.issn.1002-2694.2012.04.023

[ref71] Yang K. (2022). Investigation and analysis of risk factors of death in patients with epidemic hemorrhagic fever. Nurs. Pract. Res. 19, 200–203. doi: 10.3969/j.issn.1672-9676.2022.02.009

[ref72] Ye P. (2003). Clinical nursing of secondary infection of hemorrhagic fever with severe and critical renal syndrome. Shanghai journal of. Prev. Med. 15:405:411. doi: 10.3969/j.issn.1004-9231.2003.08.025

[ref73] Zhang J. Zheng X. Wang X. Xie F. (2015). The clinical analysis of 460 cases of hemorrhagic fever with renal syndrome complicated by multiple organ dysfunction syndrome. J. North Sichuan Med. Coll. 30, 626–629. doi: 10.3969/j.issn.1005-3697.2015.05.13

[ref74] Zhu W. Zhang X. Ran X. Hu X. Xu R. (2014). Analysis of prognostic factors affecting 202 cases of hemorrhagic fever with severe renal syndrome. Chin. J. Clin. Res. 27, 299–301. doi: 10.13429/j.cnki.cjcr.2014.03.019

[ref75] Zong R. Li C. (2001). Detection and analysis of epidemic hemorrhagic fever complicated by liver damage. Heihe Sci. Technol. 3:53.

[ref76] Zou L. X. Chen M. J. Sun L. (2016). Haemorrhagic fever with renal syndrome: literature review and distribution analysis in China. Int. J. Infect. Dis. 43, 95–100. doi: 10.1016/j.ijid.2016.01.003 26791541

[ref77] Zuo S. Q. Fang L. Q. Zhan L. Zhang P. H. Jiang J. F. Wang L. P. . (2011). Geo-spatial hotspots of hemorrhagic fever with renal syndrome and genetic characterization of Seoul variants in Beijing, China. PLoS Negl. Trop. Dis. 5:e945. doi: 10.1371/journal.pntd.0000945, PMID: 21264354 PMC3019113

