# Supplementary material for: Epidemiological and clinical characteristics of death from hemorrhagic fever with renal syndrome: a meta-analysis
Source: Front Microbiol. 2024 Apr 4;15:1329683. doi: 10.3389/fmicb.2024.1329683 (PMC11024303; doi:10.3389/fmicb.2024.1329683)
Supplement: Supplementary file 1 [file Data_Sheet_1.docx]

Table S1 Differences in personal history and clinical manifestations between patients who died and those who survived.

| Variables | OR / SMD | 95%CI | | P value |
| --- | --- | --- | --- | --- |
| age (years) | 0.478^b^ | 0.21 | 0.745 | < 0.001 |
| > 60 years | 2.409^a^ | 1.895 | 3.063 | < 0.001 |
| smoking | 1.357^a^ | 1.030 | 1.787 | 0.030 |
| onset to hospital stay | 0.391^b^ | -0.521 | -0.260 | < 0.001 |
| diabetes | 1.991^a^ | 1.080 | 3.670 | 0.027 |
| hypertension | 2.811^a^ | 2.009 | 3.933 | < 0.001 |
| MODS | 76.93^a^ | 12.689 | 466.432 | < 0.001 |
| shock | 24.075^a^ | 8.941 | 64.825 | < 0.001 |
| overlapping disease courses | 4.412^a^ | 1.744 | 11.158 | 0.002 |
| cerebral edema | 12.566^a^ | 4.428 | 35.659 | < 0.001 |
| toxic encephalopathy | 9.71^a^ | 2.847 | 33.12 | < 0.001 |
| brain haemorrhage | 88.652^a^ | 31.454 | 249.863 | < 0.001 |
| twitch | 9.341^a^ | 1.721 | 50.397 | 0.010 |
| arrhythmia | 4.337^a^ | 1.046 | 18.307 | 0.043 |
| heart failure | 5.55^a^ | 3.39 | 9.11 | < 0.001 |
| dyspnea | 2.916^a^ | 1.377 | 6.176 | 0.005 |
| ARDS | 19.068^a^ | 8.105 | 44.86 | < 0.001 |
| lung infections | 3.58^a^ | 1.399 | 9.158 | 0.008 |
| liver injury | 3.905^a^ | 1.98 | 7.703 | < 0.001 |
| gastrointestinal bleeding | 2.784^a^ | 1.602 | 4.839 | < 0.001 |
| urine protein | 2.117^a^ | 1.602 | 4.839 | < 0.001 |
| WBC | 0.717^b^ | 0.279 | 1.154 | 0.001 |
| platelet | -1.072^b^ | -1.806 | -0.377 | 0.004 |
| lactate dehydrogenase | 2.015^b^ | 0.524 | 3.506 | 0.008 |
| AST | 1.067^b^ | 0.645 | 1.490 | < 0.001 |
| ALT | 0.829^b^ | 0.258 | 0.936 | 0.002 |
| PT | 1.145^b^ | 0.426 | 1.827 | < 0.001 |
| APTT | 1.154^b^ | 0.436 | 1.873 | 0.002 |
| albumin | -0.574^b^ | -0.855 | -0.293 | < 0.001 |
| chloride ion | -0.449^b^ | -0.875 | -0.024 | 0.038 |
| fibrinogen | -0.626^b^ | -0.8285 | -0.242 | < 0.001 |

MODS: multiple organ dysfunction syndrome, ARDS: acute respiratory distress syndrome, WBC: white blood cell counts, AST: aspartate aminotransferase, ALT: alanine aminotransferase, PT: prothrombin time, APTT: activated partial thromboplastin time

^a^ OR value, ^b^ SMD value
