# Supplementary material for: Epidemiological and clinical characteristics of death from hemorrhagic fever with renal syndrome: a meta-analysis
Source: Front Microbiol. 2024 Apr 4;15:1329683. doi: 10.3389/fmicb.2024.1329683 (PMC11024303; doi:10.3389/fmicb.2024.1329683)

Figure S1. Forest plot and funnel plots of sex of HFRS patients.

a

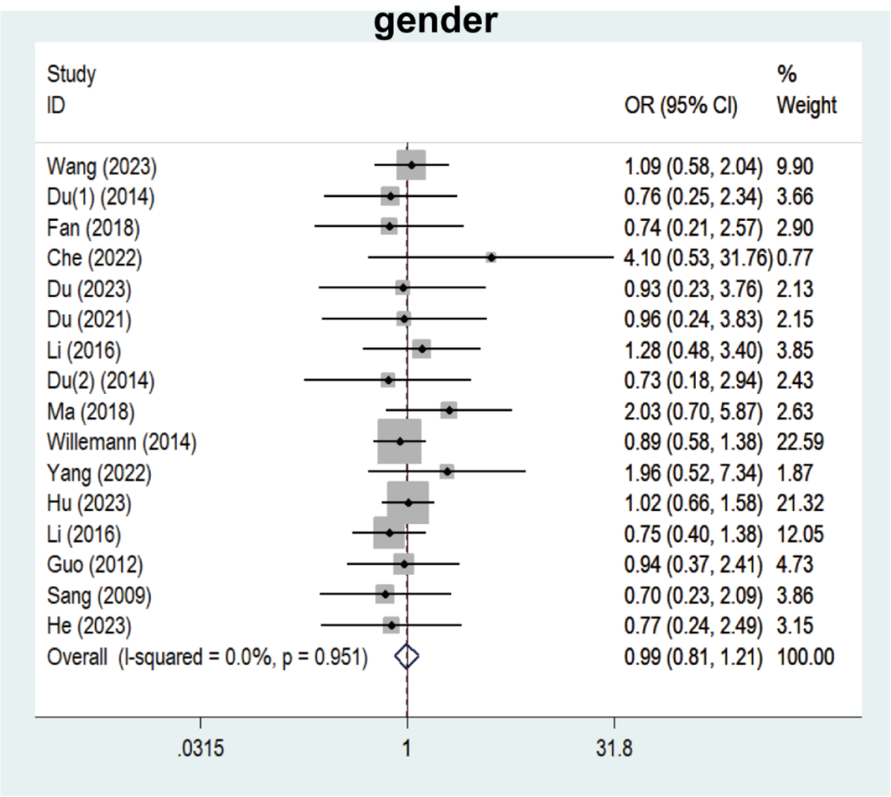

b

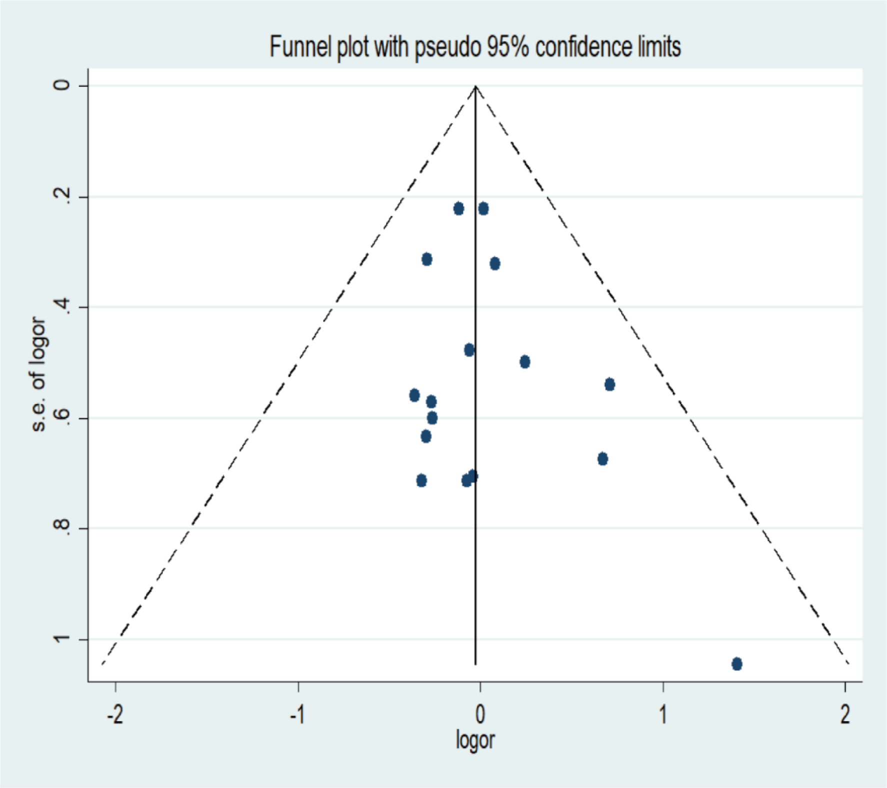

Figure S2. Forest plots of age of HFRS patients.

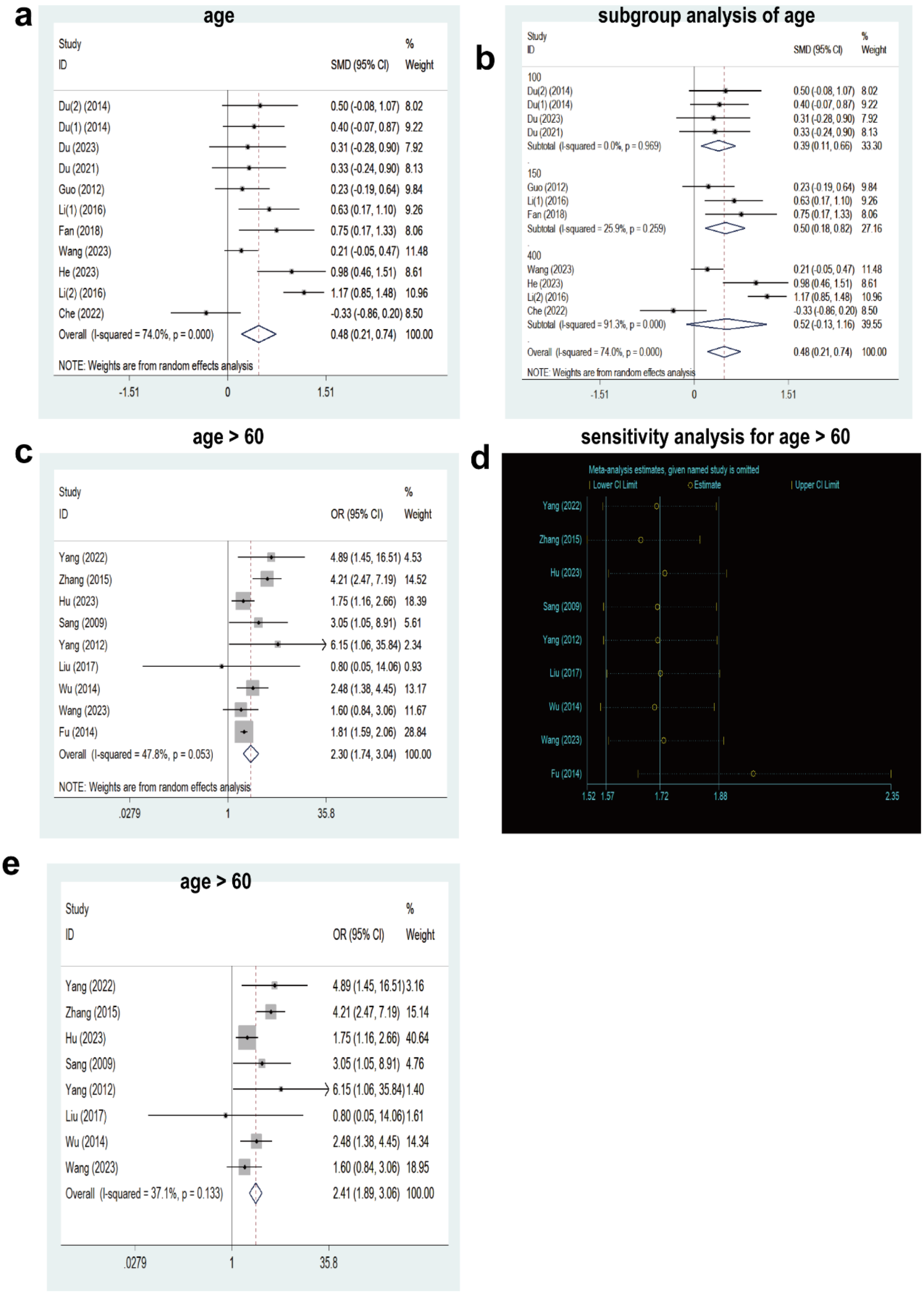

Figure S3. Forest plots of epidemiological and personal history of HFRS patients.

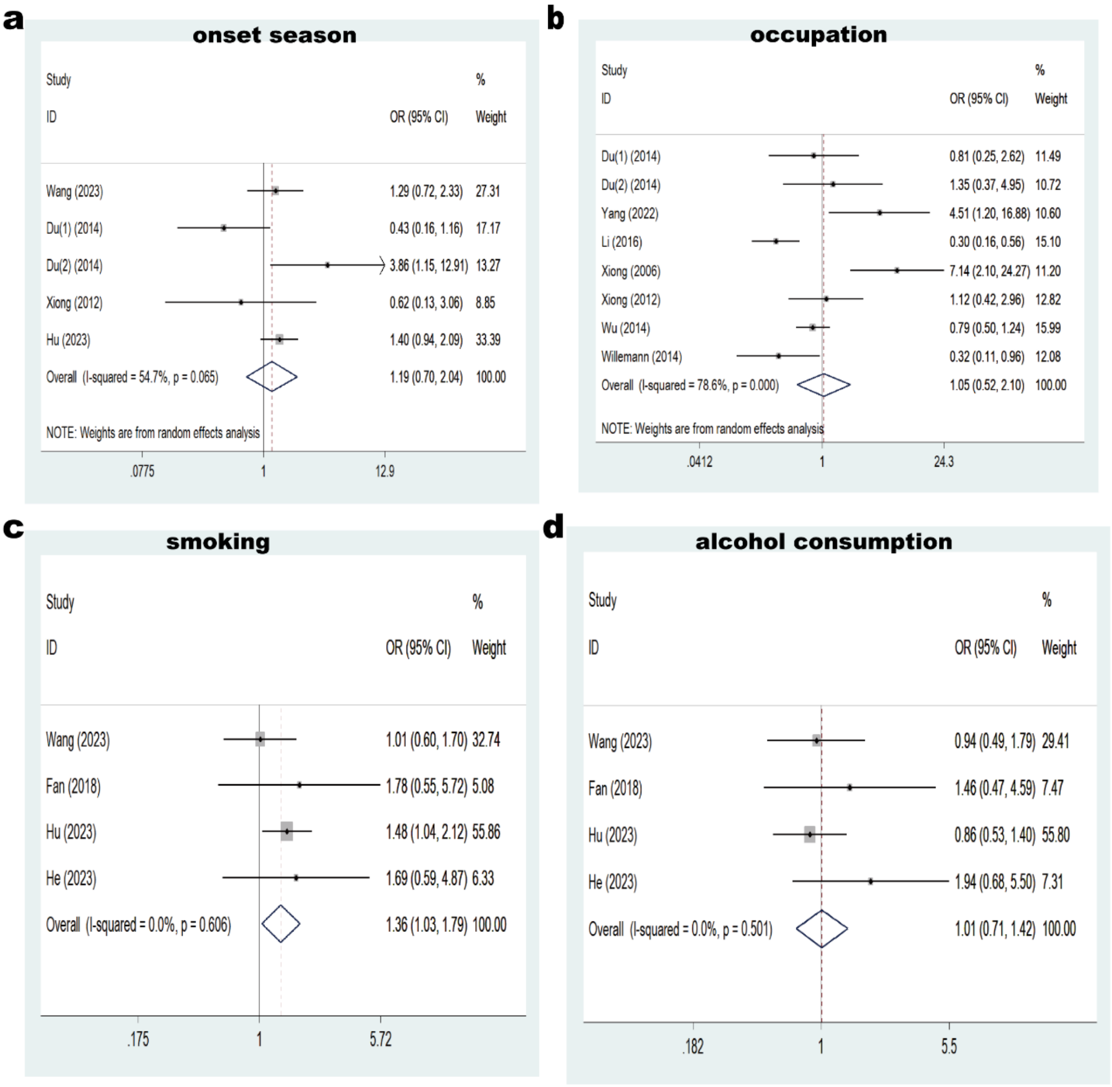

Figure S4. Forest plots of hospital admission of HFRS patients.

a

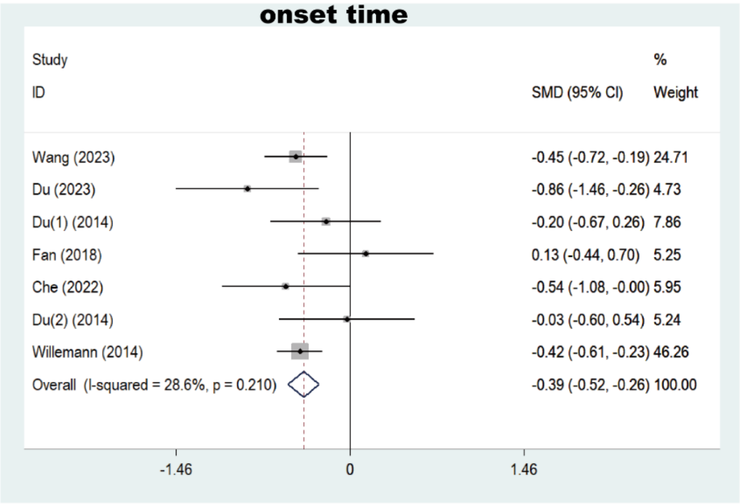

b

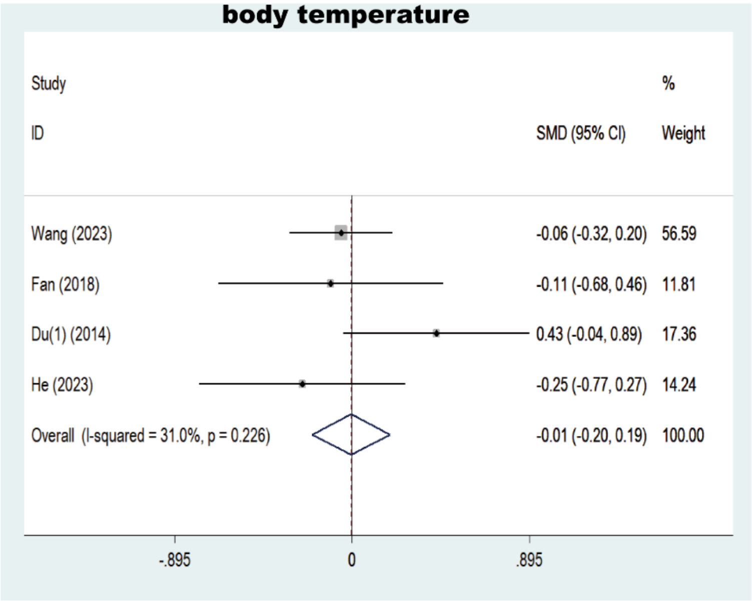

Figure S5. Forest plots of comorbidities of HFRS patients.

a

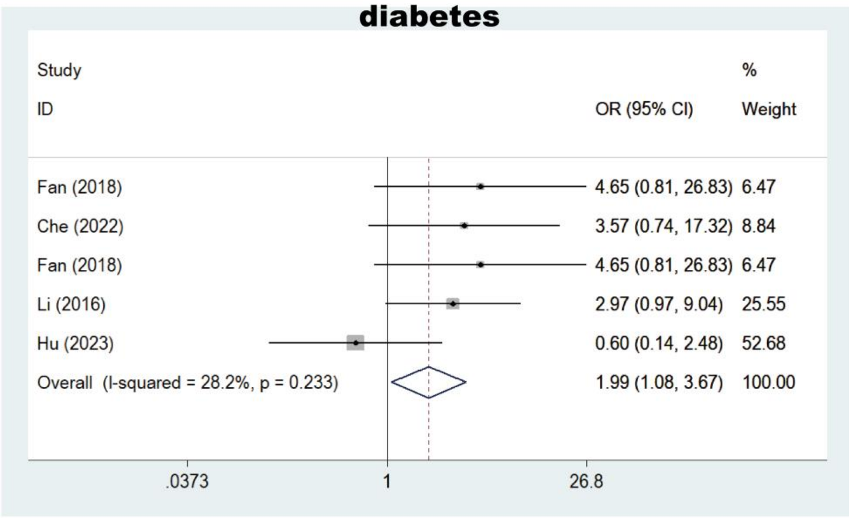

b

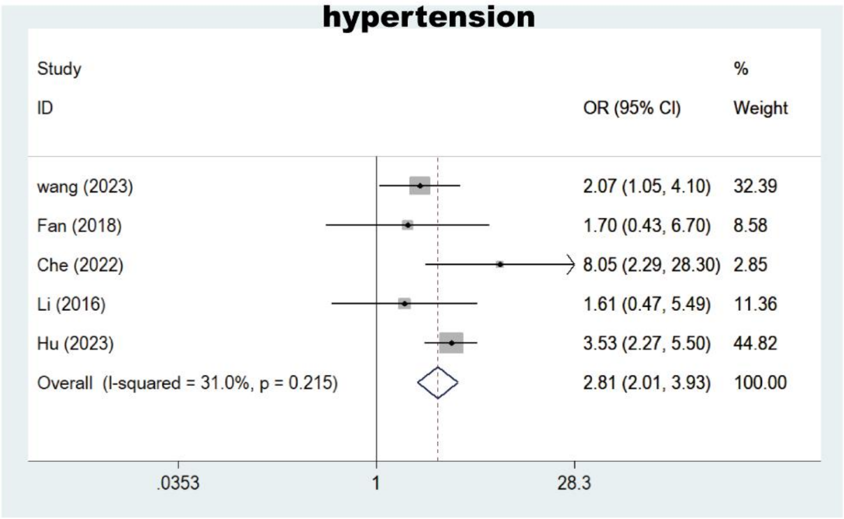

c

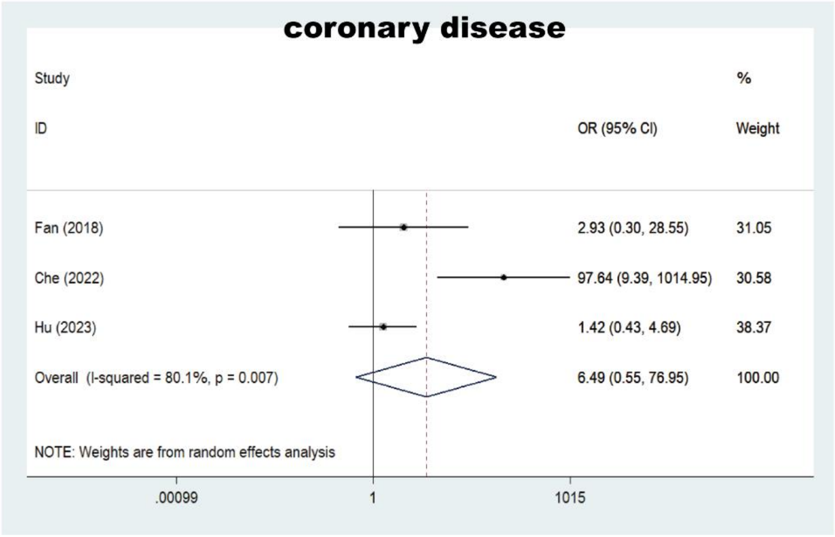

Figure S6. Forest plots of clinical manifestations of HFRS patients. (a) multiple organ dysfunction syndrome, (b) shock, (c) occurrence of disease course overlap, and (d) bacteremia.

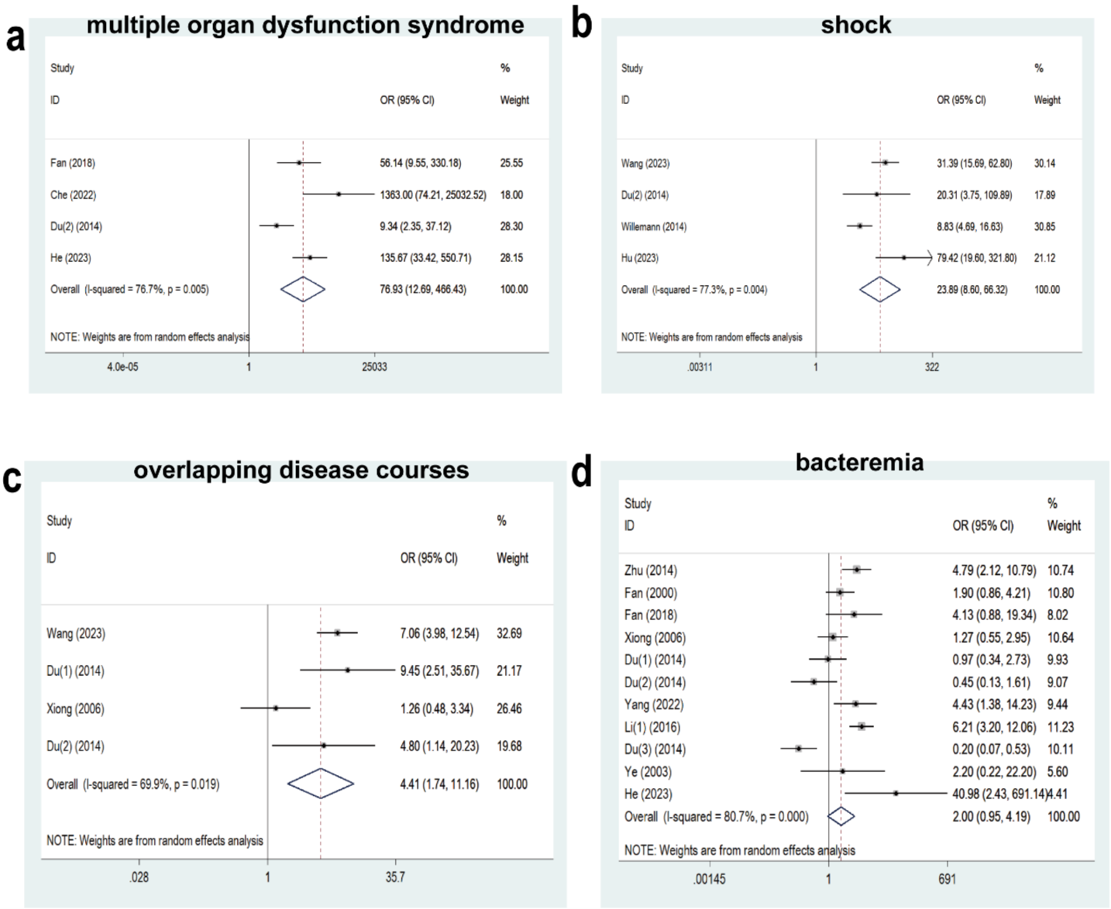

Figure S7. Forest plots of craniocerebral injury of HFRS patients. (a) cerebral edema, (b) toxic encephalopathy, (c) craniocerebral hemorrhage, and (d) convulsions.

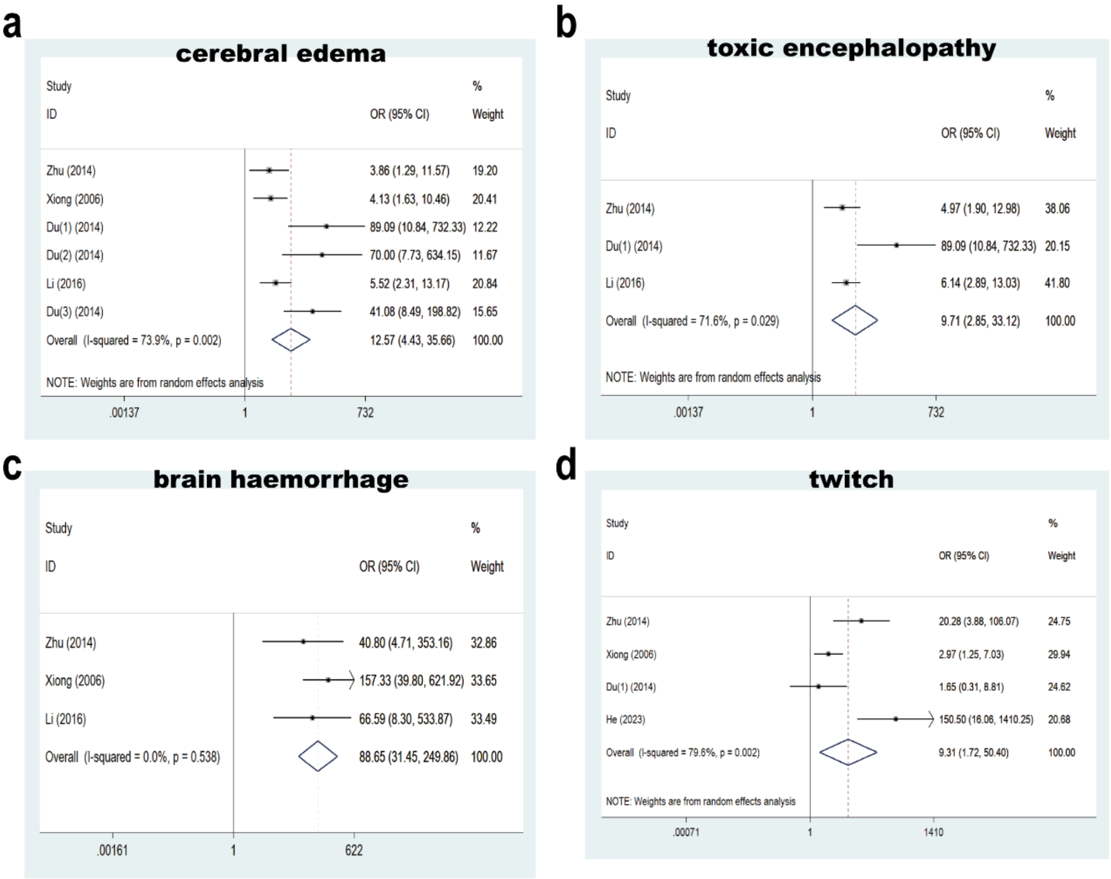

Figure S8. Forest plots of cardiac injury of HFRS patients. (a) arrhythmias, and (b) heart failure.

a

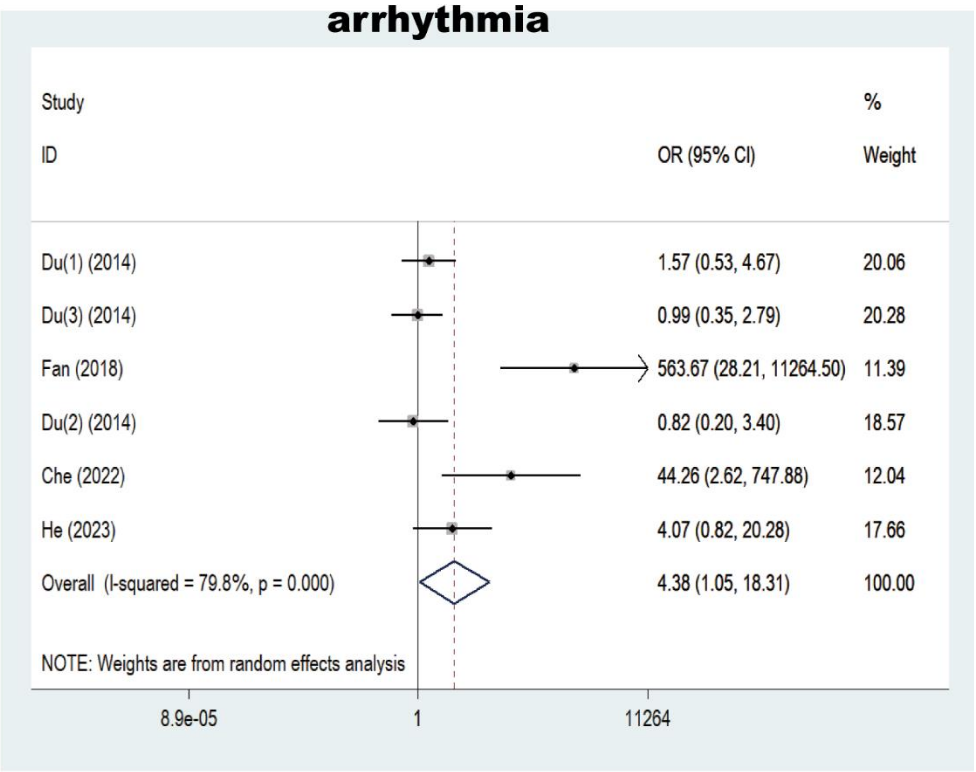

b

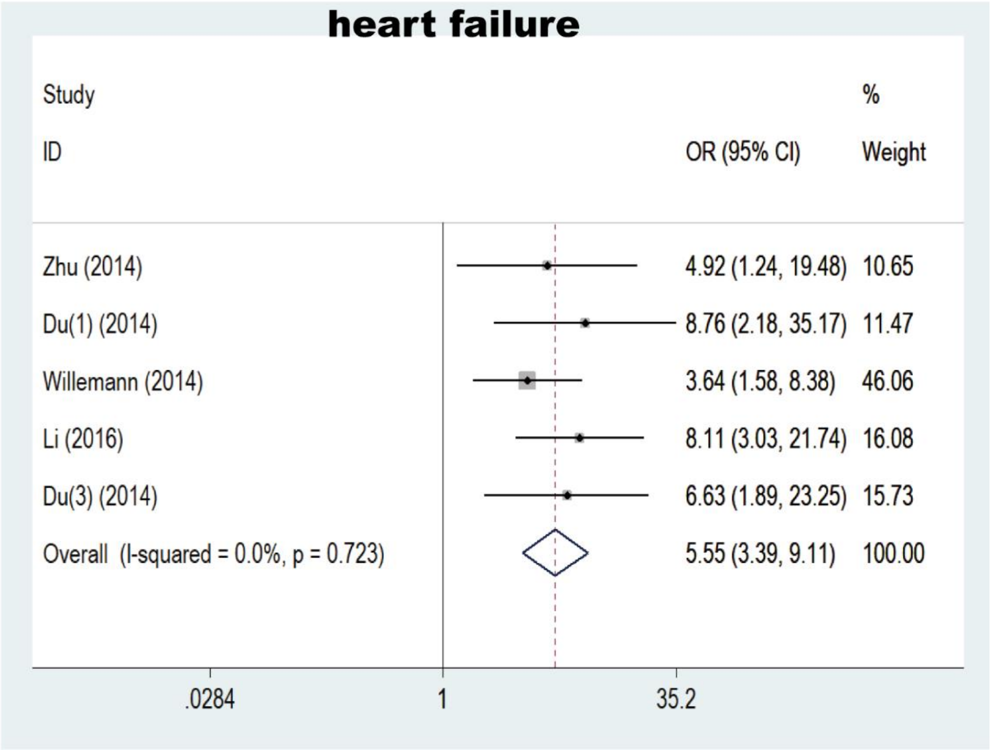

Figure S9. Forest plots of concurrent lung injury of HFRS patients. (a) dyspnea, (b) acute respiratory distress syndrome, and (c) lung infections.

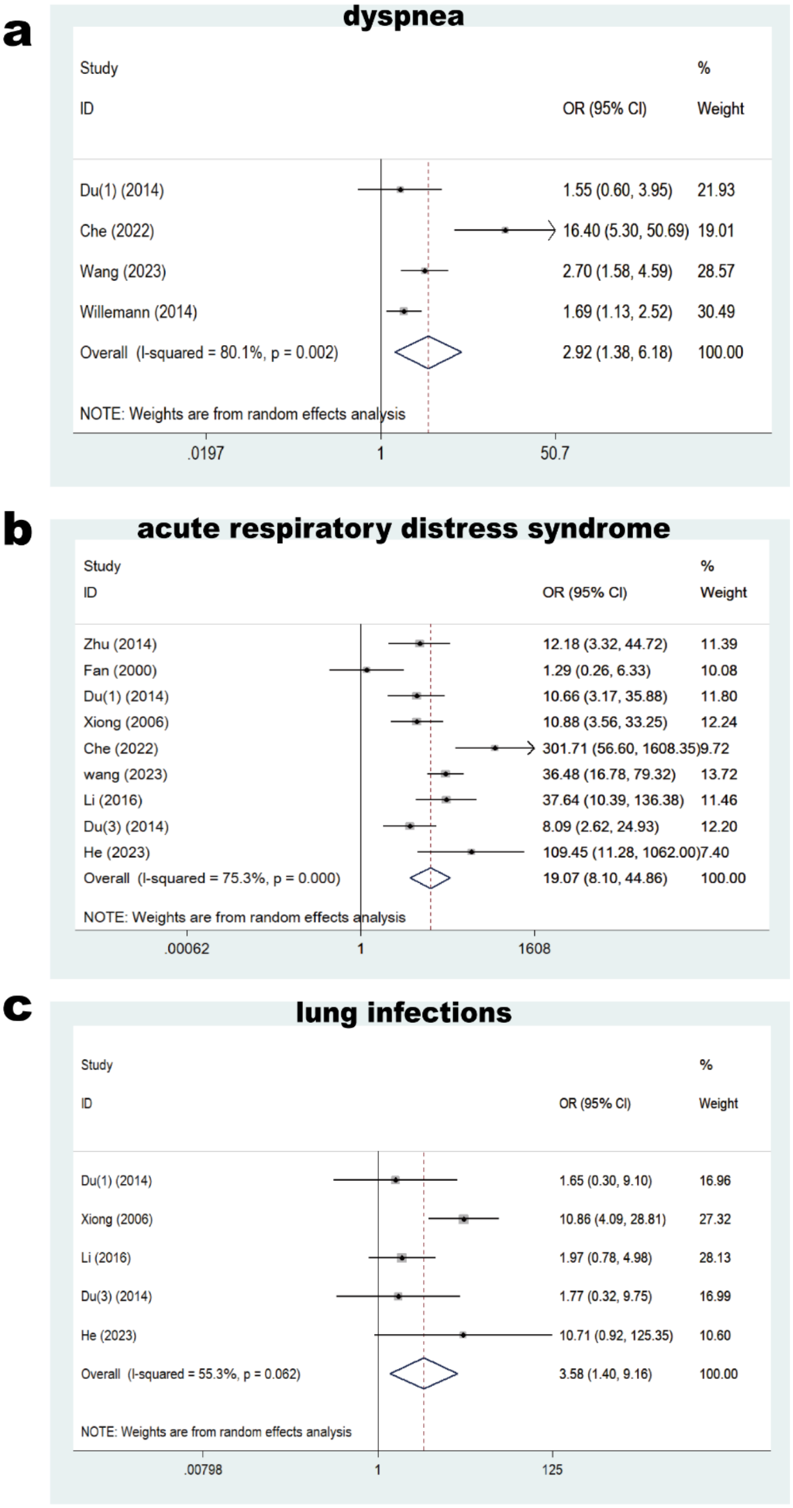

Figure S10. Forest plots of digestive complications of HFRS patients. (a) liver injury, and (b) gastrointestinal bleeding.

a

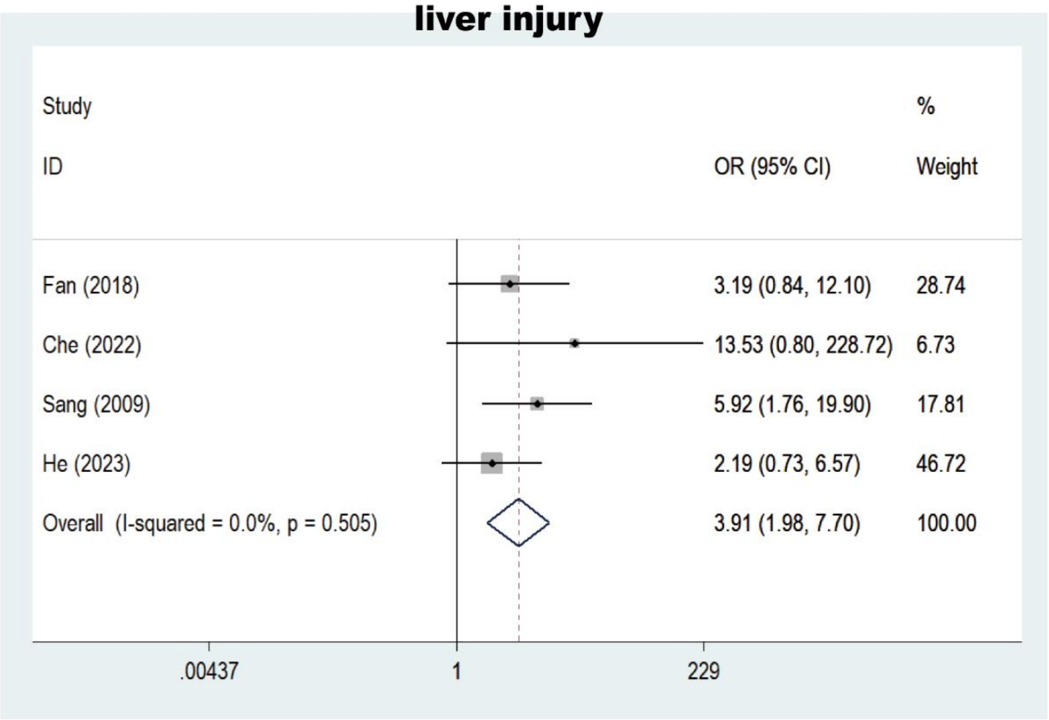

b

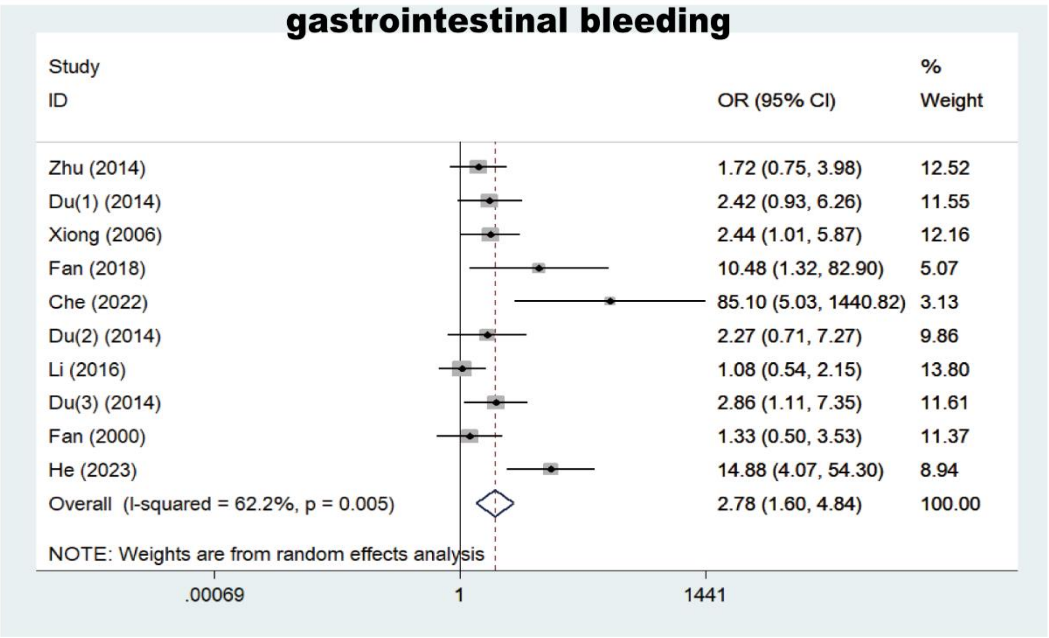

Figure S11. Forest plots of urinary system injuries of HFRS patients. (a) acute kidney injury, and (b) urine protein.

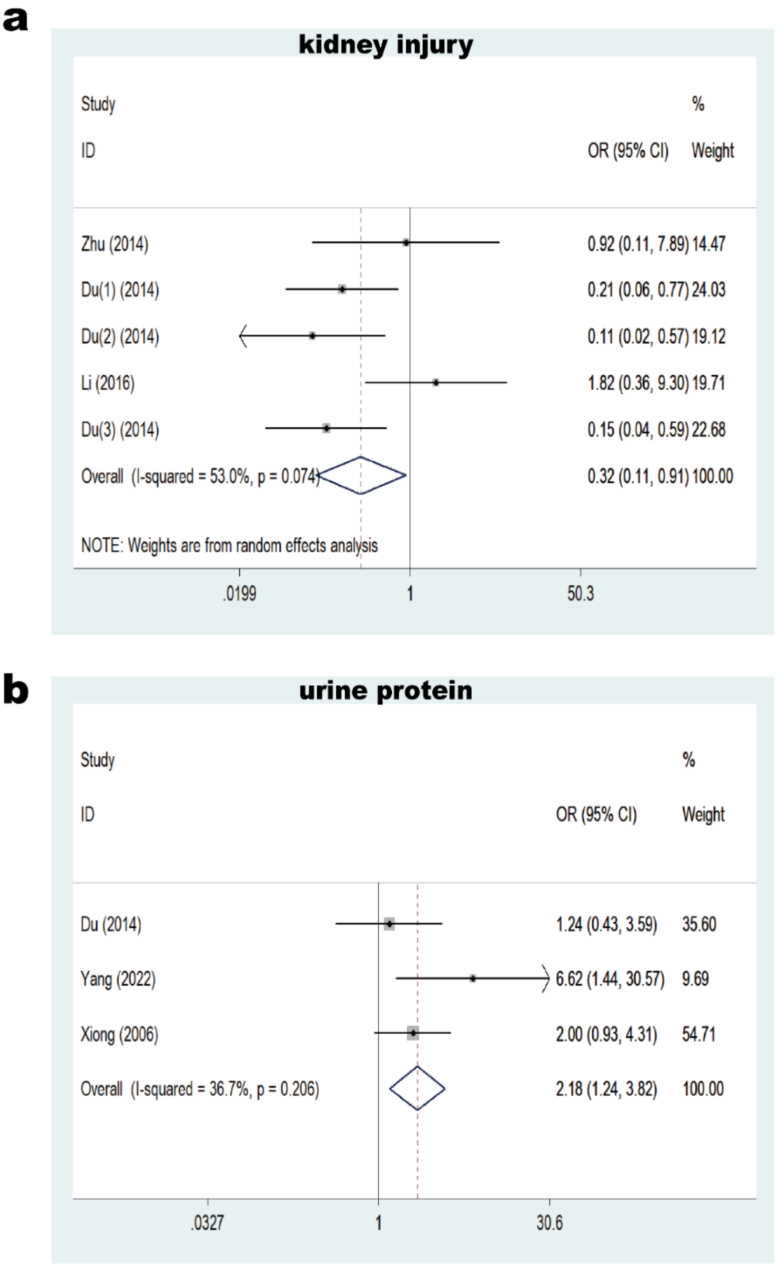

Figure S12. Forest plots of blood routine of HFRS patients. (a) white blood cell count, (b) platelet count, and (c) hemoglobin count.

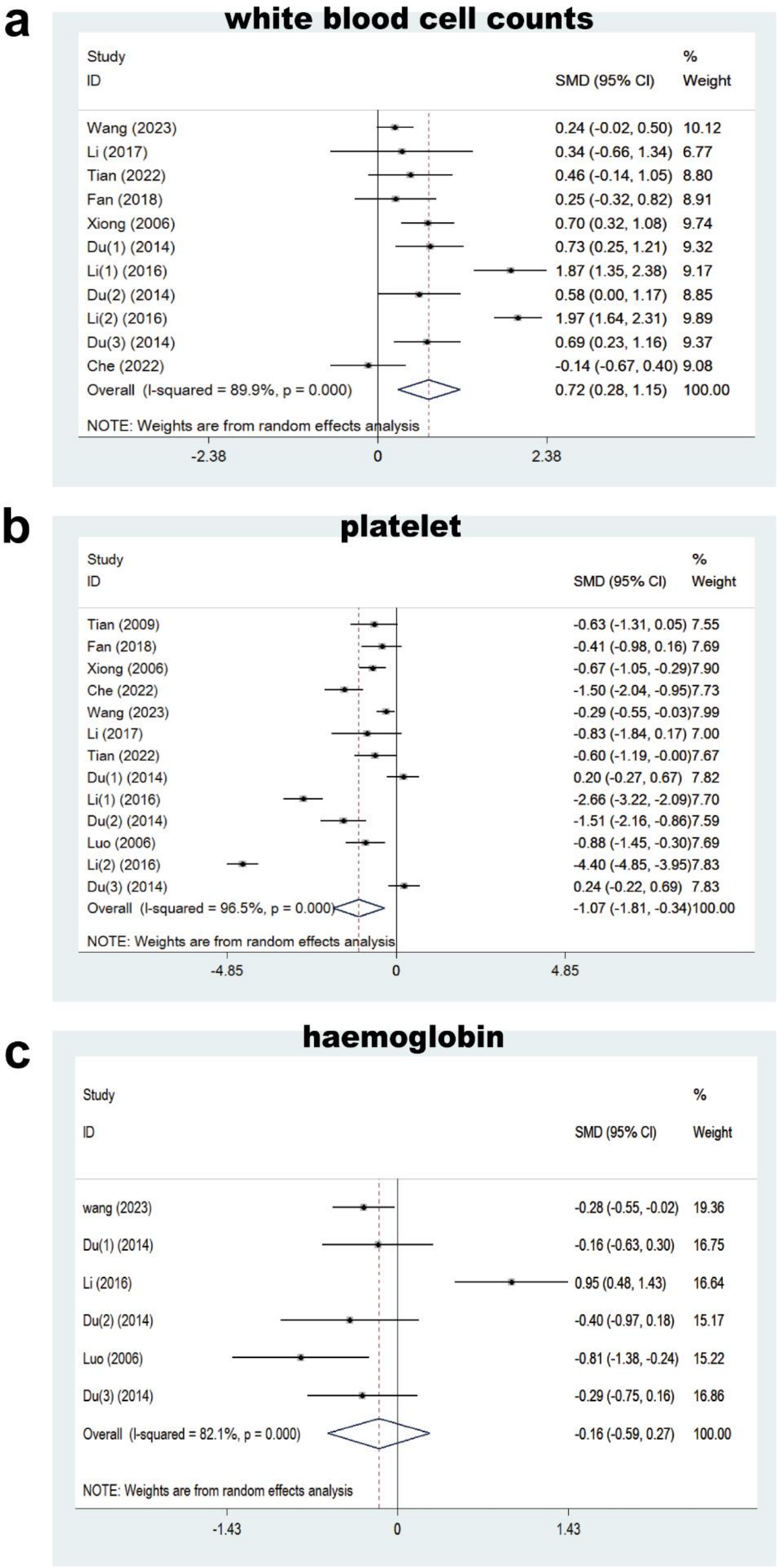

Figure S13. Forest plots of biochemical tests of HFRS patients. (a) lactate dehydrogenase, (b)aspartate transaminase, (c) alanine aminotransferase, (d) prothrombin time, (e) activated partial thromboplastin time, (f) albumin, (g)chloride ion, and (h)fibrinogen.

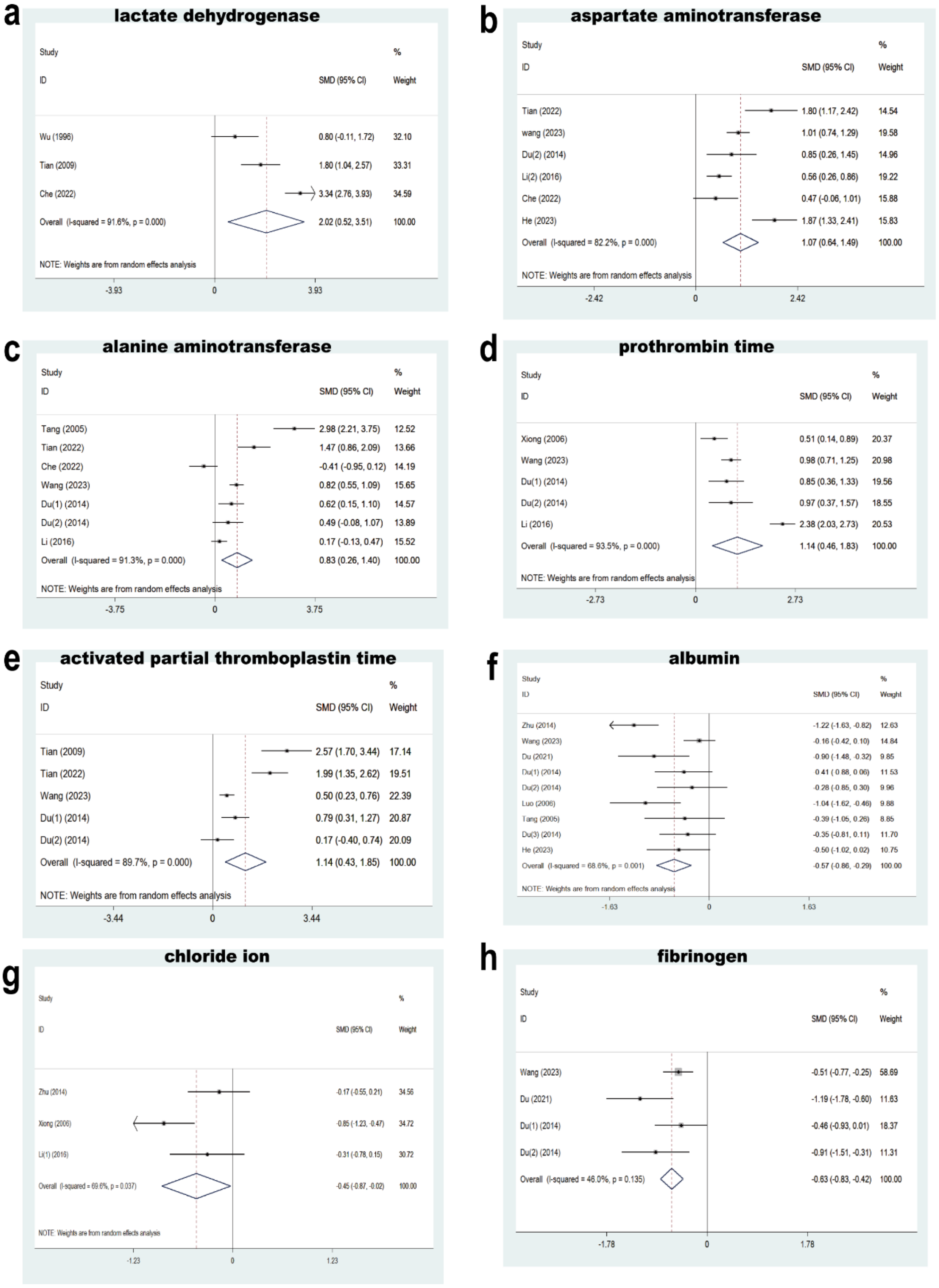

Figure S14. Forest plots of biochemical tests of HFRS patients. (a)urea nitrogen, (b)serum creatinine, (c)sodium ion, (d) potassium ion, and (e) total bilirubin.

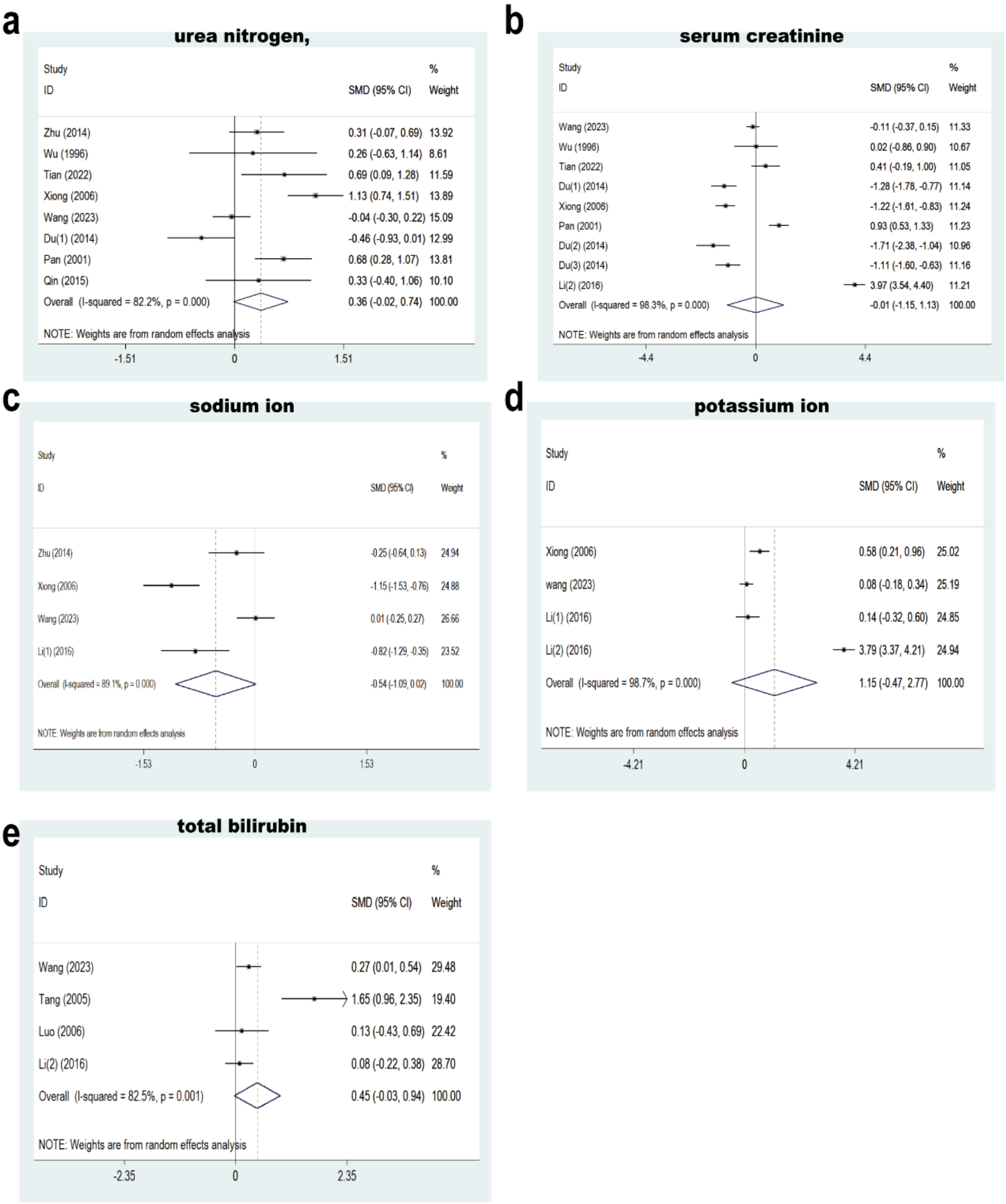

Figure S15. Forest plots of treatment of HFRS patients.

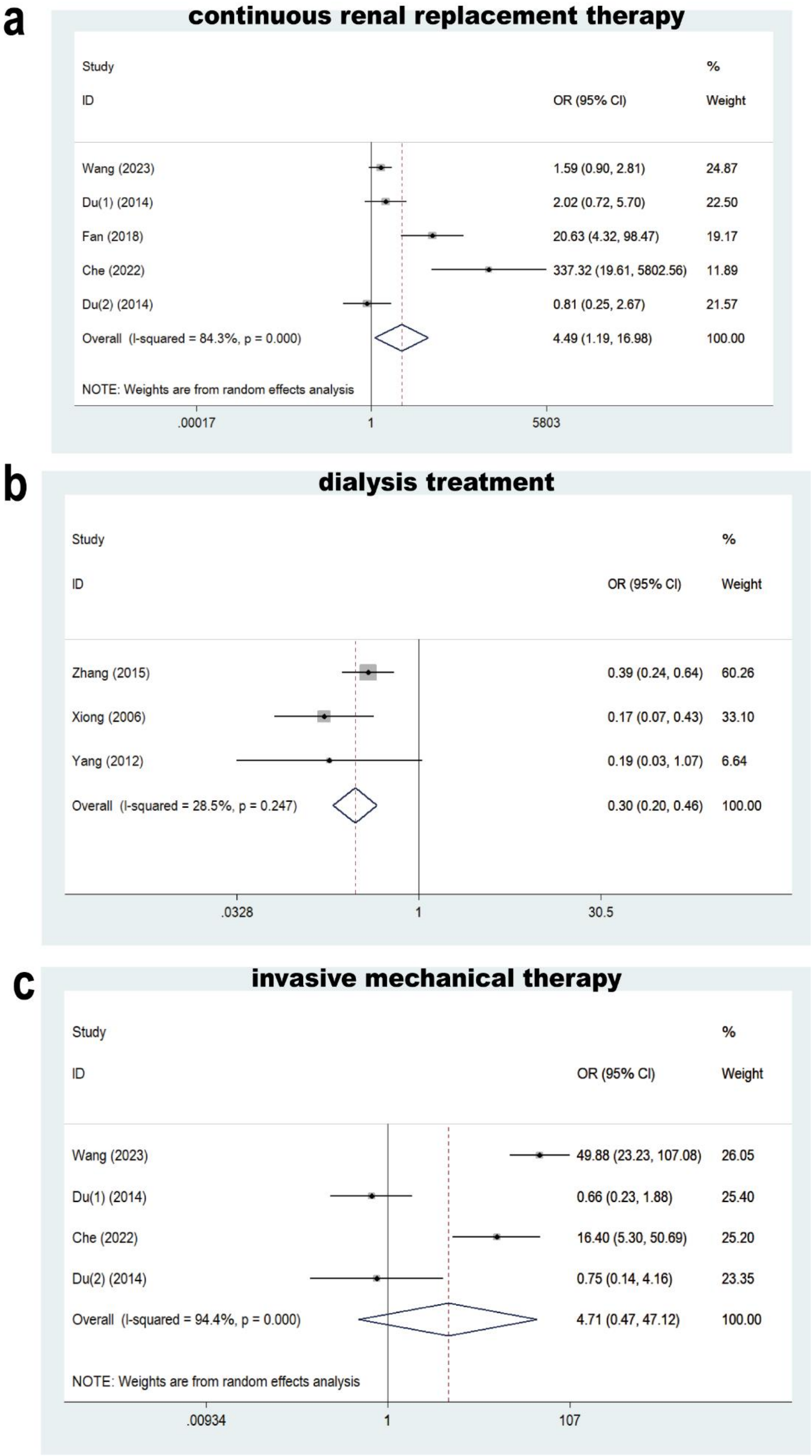

Figure S16. Forest plots of hospitalization of HFRS patients.

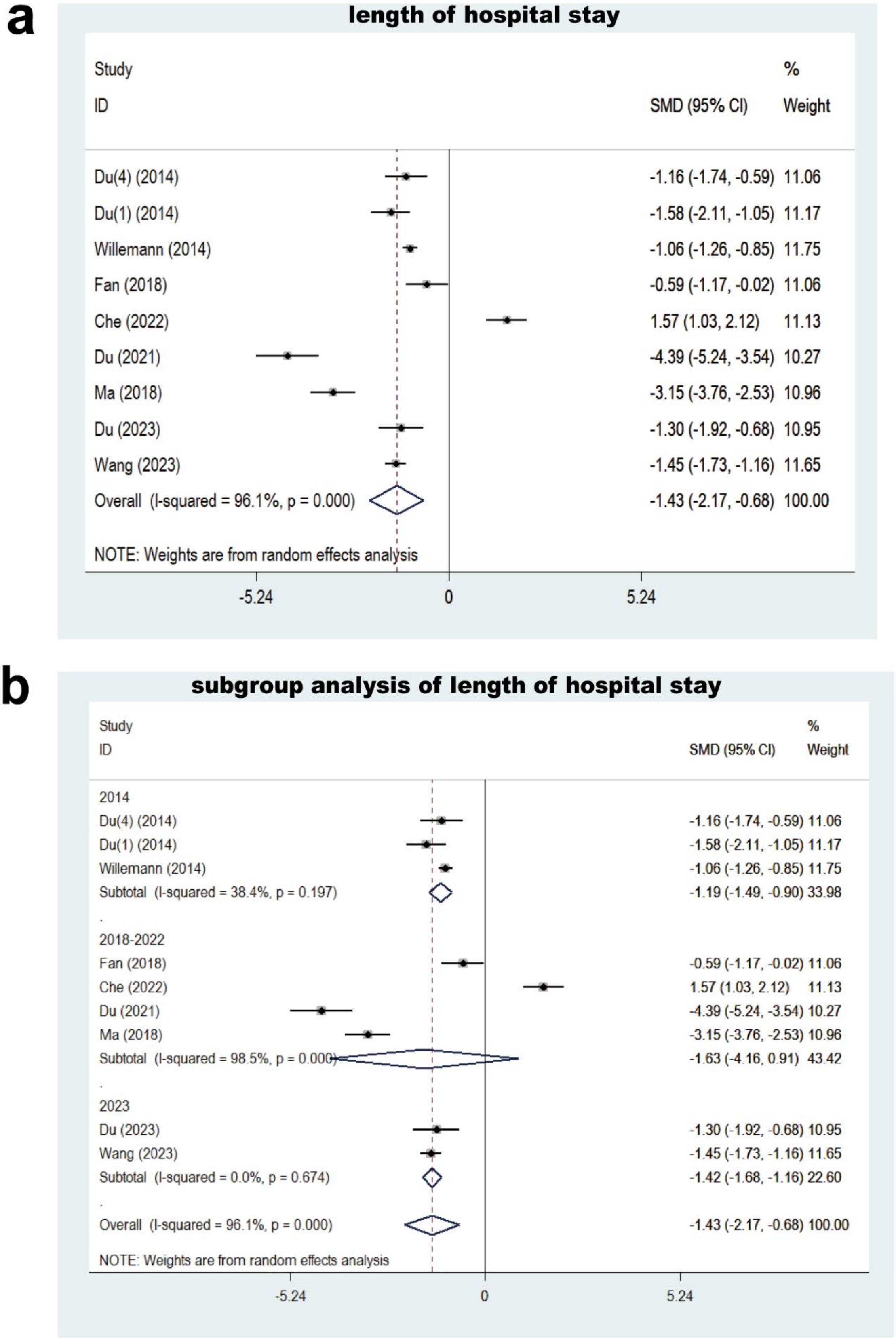

Supplement: Supplementary file 2 [file Presentation_1.pdf]
